# Evaluation of Low-Value Services Across Major Medicare Advantage Insurers and Traditional Medicare

**DOI:** 10.1001/jamanetworkopen.2024.42633

**Published:** 2024-11-01

**Authors:** Ciara Duggan, Adam L. Beckman, Ishani Ganguli, Mark Soto, E. John Orav, Thomas C. Tsai, Austin Frakt, Jose F. Figueroa

**Affiliations:** 1 Department of Health Policy and Management, Harvard T. H. Chan School of Public Health, Boston, Massachusetts; 2 Department of Medicine, Division of General Internal Medicine, Brigham and Women’s Hospital, Harvard Medical School, Boston, Massachusetts; 3 Department of Surgery, Brigham and Women’s Hospital, Harvard Medical School, Boston, Massachusetts; 4 Partnered Evidence-Based Policy Resource Center, Boston VA Healthcare System, Boston, Massachusetts; 5 Department of Health Law, Policy, & Management, Boston University School of Public Health, Boston, Massachusetts

## Abstract

**Importance:**

Compared with traditional Medicare (TM), Medicare Advantage (MA) insurers have greater financial incentives to reduce the delivery of low-value services (LVS); however, there is limited evidence at a national level on the prevalence of LVS utilization among MA vs TM beneficiaries and whether LVS utilization rates vary among the largest MA insurers.

**Objective:**

To determine whether there are differences in the rates of LVS delivered to Medicare beneficiaries enrolled in MA vs TM, overall and by the 7 largest MA insurers.

**Design, Setting, and Participants:**

This cross-sectional study included Medicare beneficiaries aged 65 years and older residing in the US in 2018 with complete demographic information. Eligible TM beneficiaries were enrolled in Parts A, B, and D, and eligible MA beneficiaries were enrolled in Part C with Part D coverage. Data analysis was conducted between February 2022 and August 2024.

**Exposures:**

Medicare plan type.

**Main Outcomes and Measures:**

The primary outcome was utlization of 35 LVS defined by the Milliman Health Waste Calculator. An overdispersed Poisson regression model was used to calculate estimated margins comparing risk-adjusted rates of LVS in TM vs MA, overall and across the 7 largest MA insurers.

**Results:**

The study sample included 3 671 364 unique TM beneficiaries (mean [SD] age, 75.7 [7.7] years; 1 502 631 female [40.9%]) and 2 299 618 unique MA beneficiaries (mean [SD] age, 75.3 [7.3] years; 983 592 female [42.8%]). LVS utilization was lower among those enrolled in MA compared with TM (50.02 vs 52.48 services per 100 beneficiary-years; adjusted absolute difference, −2.46 services per 100 beneficiary-years; 95% CI, −3.16 to −1.75 services per 100 beneficiary-years; *P* < .001). Within MA, LVS utilization was lower among beneficiaries enrolled in HMOs vs PPOs (48.03 vs 52.66 services per 100 beneficiary-years; adjusted absolute difference, −4.63 services per 100 beneficiary-years; 95% CI, −5.53 to −3.74 services per 100 beneficiary-years; *P* < .001). While MA beneficiaries enrolled in UnitedHealth, Humana, Centene, and smaller MA insurers had lower rates of LVS compared with those in TM, beneficiaries enrolled in CVS, Cigna, and Anthem showed no differences. Blue Cross Blue Shield Association plans had higher rates of LVS compared with TM.

**Conclusions and Relevance:**

In this cross-sectional study of nearly 6 million Medicare beneficiaries, utilization of LVS was on average lower among MA beneficiaries compared with TM beneficiaries, possibly owing to stronger financial incentives in MA to reduce LVS; however, meaningful differences existed across some of the largest MA insurers, suggesting that MA insurers may have variable ability to influence LVS reduction.

## Key Points

**Question:**

Do rates of low-value service (LVS) utilization differ between traditional Medicare (TM) and the largest Medicare Advantage (MA) insurers?

**Findings:**

In this cross-sectional study of 3 671 364 TM beneficiaries and 2 299 618 MA beneficiaries, LVS utilization was lower among MA beneficiaries compared with TM beneficiaries, but there was substantial variation in the use of LVS across 7 of the largest MA insurers, with some MA insurers not having lower rates than TM; within MA plans, LVS utilization was also lower among those enrolled in health maintenance organizations compared with preferred provider organizations.

**Meaning:**

These findings suggest that there is substantial heterogeneity in the use of low-value care across the major MA insurer organizations, and not all outperform TM.

## Teaser

This cross-sectional study compares rates of low-value service utilization among beneficiaries enrolled in traditional Medicare and various Medicare Advantage insurers.

## Introduction

As of 2024, more than one-half of Medicare beneficiaries are enrolled in the rapidly growing Medicare Advantage (MA) program, the private managed care option that serves as an alternative to traditional fee-for-service Medicare (TM).^1^ MA insurers receive monthly capitated payments from the Centers for Medicare & Medicaid Services (CMS) to cover hospital, outpatient, postacute, and long-term care for Medicare beneficiaries, as well as some additional supplemental benefits. As a result, MA insurers have strong incentives to limit potentially wasteful spending, including the use of low-value services (LVS) that provide little or no clinical benefit to beneficiaries yet remain a persistent source of inefficiency and potential harm across the US health care system.^2,3^

Despite longstanding efforts to curb their use, older adults in Medicare continue to receive LVS.^3,4,5,6,7,8,9,10^ However, evidence regarding differences in LVS use among MA vs TM beneficiaries remains limited. Previous studies comparing low-value care utilization in MA and TM have been limited by small sample size^10^ or by reliance on data from a single MA insurer.^11^ Thus, the extent to which there may be meaningful differences in the use of LVS between MA and TM at a national level is not clear. Substantial variation may also exist across different MA insurers, which could stem from plan differences in insurance benefit design, copayments, and prior authorization requirements.^12,13^ Because MA is now the dominant form of Medicare nationally and continues growing, understanding potential variations in the use of LVS is increasingly important as a way of understanding quality differences between MA vs TM and heterogeneity within MA itself.

Therefore, using national Medicare data, this study answered the following key questions. First, are there significant differences in the use of LVS between Medicare beneficiaries enrolled in MA vs TM at a national level? Second, are these differences consistent across major MA parent insurer companies?

## Methods

This cross-sectional study was reviewed by the institutional review board at the Harvard T.H. Chan School of Public Health, which waived informed consent of study participants given that data used was aggregated and deidentified. The study follows the Strengthening the Reporting of Observational Studies in Epidemiology (STROBE) reporting guideline for observational studies.

### Data and Study Sample

We used a 20% random sample of TM administrative claims data and a 20% random sample of MA, or Part C, encounter data from 2018. We limited our sample to include beneficiaries aged 65 years and older residing in the US with complete demographic information who were enrolled in Parts A, B, and D for all months alive during the year among TM beneficiaries or Part C with Part D coverage for MA beneficiaries (eAppendix 1 in Supplement 1). We further excluded beneficiaries who switched between TM and MA at any point midyear, thus limiting the sample to those continuously enrolled in either MA or TM. Given concerns about the incompleteness of certain MA contracts in encounter data, we limited analyses to MA beneficiaries in highly reliable and complete MA contracts using a previously validated approach (eAppendix 2 in Supplement 1).^14,15,16^

Given our interest in assessing variation across different MA insurers, we obtained parent organization and enrollment data for highly reliable MA contracts from the plan characteristics file. We grouped contracts by major insurer organization and identified the 7 largest insurer groups represented in our sample based on the number of beneficiaries enrolled in 2018, which included UnitedHealth, Humana, CVS, Blue Cross Blue Shield (BCBS) Association plans (grouped together for simplicity and given that the BCBS Association is a national association that owns and manages the BCBS trademarks across the country), Cigna, Centene, and Anthem. We categorized MA beneficiaries in the sample into these 7 major parent insurer groups while grouping the remaining beneficiaries (eg, those enrolled in MA plans offered by smaller insurers) into an other MA insurers category.

### Primary Outcomes

For our primary outcomes, we operationalized LVS definitions described in the MedInsight Health Waste Calculator version 8.0 (Milliman), a proprietary software program that identifies potentially low-value services as defined by the Choosing Wisely campaign and US Preventive Task Force.^17^ Three physician reviewers (J.F.F., I.G., and T.C.T.) reviewed the available measures described by the calculator and identified 35 unique Milliman LVS definitions relevant to the Medicare population. These services included 3 common medical treatments, 11 services related to imaging, 6 services related to diagnostic and preventative testing, 4 services related to cardiovascular testing, 4 services related to specific procedures and surgeries, 4 services related to preoperative evaluations, and 3 cancer screening services. The calculator identifies services as necessary, likely to be wasteful, and wasteful; we only included services that met the wasteful criteria to be conservative. We excluded LVS in January through March 2018 to allow at least 3 months of claims history to determine appropriate clinical exclusions of LVS. For more details, see eAppendix 3 in Supplement 1. We identified beneficiaries at risk for each LVS using the Milliman calculator and counted eligible beneficiary-months from April 1, 2018, until death or December 31st, 2018, for each measure.

### Covariates

We used the Medicare Beneficiary Summary File to identify beneficiary demographics, including age, sex, race and ethnicity, dual eligibility, and low-income subsidy (LIS) status. For race and ethnicity, we used the Research Triangle Institute race code, which groups beneficiaries into Black, Hispanic, White, and other (due to sample size, we grouped American Indian and Alaska Native, Asian and Pacific Islander, and other [ie, any race or ethnicity not otherwise specified] categories into 1 group)^18^; race and ethnicity were included to account for documented racial and ethnic disparities in the receipt of low-value care among Medicare beneficiaries.^19,20^ We identified beneficiaries with at least 1 month of Medicaid eligibility as dual-eligible and identified beneficiaries as non-, partial, or full LIS recipients based on their LIS status in January 2018.

To identify a proxy for patient severity, we calculated the CMS hierarchical condition category (HCC) risk score for all beneficiaries based on 2018 inpatient and outpatient data for both MA and TM beneficiaries. We excluded chart review claims from MA encounter records as done in prior studies given that the phenomenon of inflated coding may be particularly evident in these records.^21,22,23,24^ In addition, for primary analyses of MA beneficiaries, we deflated HCC risk scores of all MA beneficiaries by 11% based on prior literature showing that MA plans generate anywhere between 6% to 16% more intensive coding than TM.^25^

We additionally identified rurality, census region, and Area Deprivation Index values (ADI) for each beneficiary. We identified rural counties as those with a 2013 rural-urban continuum code greater than 7. Using zip code of residence, we identified the 2019 ADI, which is constructed from the previous 5 years of data (2015-2019) from the American Community Survey.^26^

### Statistical Analyses

We first compared demographics of beneficiaries enrolled in highly reliable MA contracts with those in TM, as well as the MA beneficiaries enrolled in the less reliable and excluded contracts using standardized mean differences (SMDs). SMDs less than 0.10 were considered negligible differences.

Next, we performed a series of overdispersed Poisson regression models to determine the adjusted differences in LVS utilization per 100 beneficiary-years among MA vs TM beneficiaries. Beneficiaries were the unit of analysis, and the number of LVS during 2018 (calculated from eligible beneficiary-months) was the outcome variable. For presentation of results, we multiplied the model estimate of the number of LVS by 100 to reflect the number of LVS per 100 beneficiary-years. An offset was included in the models to account for months that beneficiaries were alive in 2018. We determined adjusted differences for each of the 35 LVS measures as well as differences overall and across 7 LVS categories (ie, common medical treatments, imaging, diagnostic and preventative testing, cardiovascular testing, specific procedures and surgeries, preoperative evaluations, and cancer screening). Estimated rates of LVS utilization and absolute differences between MA and TM were calculated using estimated margins. The primary exposure was Medicare insurance program (MA vs TM) enrollment, and the adjustment covariates included age, sex, race and ethnicity, dual eligibility status, and HCC risk score. Models included hospital referral region (HRR) fixed-effects and robust standard errors clustered within HRRs to account for potential within-HRR correlation. A 2-sided *P* < .05 was considered significant and was not adjusted for multiple outcomes given the exploratory nature of the research.

Next, given that health maintenance organizations (HMOs) are more restrictive than preferred provider organizations (PPOs) and have been previously shown to be associated with lower rates of service utilization,^27,28^ we repeated our primary overdispersed Poisson models as described previously to determine the adjusted differences in LVS utilization among MA beneficiaries in HMOs vs those in PPOs. Given the hypothesis that meaningful variation exists across major MA insurers, we also repeated our primary analyses comparing LVS utilization across the 7 largest MA parent insurer groups and the group of other smaller insurers relative to TM.

We tested our assumptions with several sensitivity analyses. First, we repeated our models after deflating HCC risk scores among MA beneficiaries by 6% and 16%, which reflect the full range of potential upcoding as documented by the literature.^25^ While our main analyses excluded beneficiaries who switched between TM and MA at any point midyear, we conducted a sensitivity analysis including beneficiaries who switched during the year. For this analysis, beneficiaries were assigned to the MA or TM program based on their enrollment at the beginning of 2018. Additionally, while our main analyses excluded MA beneficiaries who were not enrolled in contracts with highly reliable and complete encounter data, we conducted a separate analysis comparing LVS rates among beneficiaries enrolled in included MA contracts compared with those enrolled in the excluded contracts. We also conducted an analysis in which we excluded decedents, limiting our sample to beneficiaries who survived the year. Given that the BCBS Association is an association of smaller, independent companies, we examined potential heterogeneity among BCBS plans by estimating LVS use separately for BCBS beneficiaries in each of the 5 states with the highest enrollment in BCBS plans with highly reliable and complete data (Michigan, Alabama, North Carolina, Arizona, and Rhode Island). We then compared LVS use in each of these BCBS states with TM beneficiaries in the same state. Finally, we repeated our main models using county fixed-effects rather than HRR fixed-effects. Data were analyzed between February 2022 and August 2024 using Stata software version 17.0 (StataCorp).

## Results

### Beneficiary Characteristics of Study Sample

The study sample included 3 671 364 unique TM beneficiaries (mean [SD] age, 75.7 [7.7] years; 1 502 631 female [40.9%]) and 2 299 618 unique MA beneficiaries (mean [SD] age, 75.3 [7.3] years; 983 592 female [42.8%]) (Table 1). A total of 1 211 788 MA beneficiaries were excluded from the sample because they were enrolled in unreliable MA contracts or were not enrolled in MA for all months alive during the year (eTable 1 in Supplement 1). MA beneficiaries included in the sample vs those excluded from the sample were similar with regards to age, sex, LIS status, HCC risk score, and urbanicity (SMD < 0.10). However, MA beneficiaries in the study sample were less likely to be dual-eligible for Medicaid and more likely to be in PPO plans than those excluded.

**Table 1.** Characteristics of Beneficiaries With Traditional Medicare vs Medicare Advantage in the Study Sample^a^

Characteristic	Beneficiaries, No. (%) (N = 5 970 982)		SMD
	Traditional Medicare (n = 3 671 364)	Medicare Advantage (n = 2 299 618)	
Age, mean (SD), y	75.7 (7.7)	75.3 (7.3)	0.05
Age group, y			
65-74	1 903 364 (51.8)	1 220 706 (53.1)	0.02
75-84	1 212 673 (33.0)	782 579 (34.0)	0.02
≥85	555 327 (15.1)	296 333 (12.9)	0.06
Sex			
Female	1 502 631 (40.9)	983 592 (42.8)	0.04
Male	2 168 733 (59.1)	1 316 026 (57.2)	0.04
Race and ethnicity			
Hispanic	184 239 (5.0)	225 273 (9.8)	0.18
Non-Hispanic Asian	111 146 (3.0)	75 546 (3.3)	0.01
Non-Hispanic Black	243 473 (6.6)	279 271 (12.1)	0.19
Non-Hispanic White	3 025 837 (82.4)	1 668 506 (72.6)	0.24
Other or unknown^b^	106 669 (2.9)	51 022 (2.2)	0.04
Dual Eligibility			
Medicare and Medicaid eligible	614 639 (16.7)	307 261 (13.4)	0.09
Medicare only	3 056 725 (83.3)	1 992 357 (86.6)	0.09
LIS status			
Non-LIS recipient	2 925 989 (79.7)	1 909 317 (83.0)	0.09
Partial LIS recipient	725 797 (19.8)	368 287 (16.0)	0.10
Full LIS recipient	19 578 (0.5)	22 014 (1.0)	0.05
Hierarchical condition category risk score, mean (SD)	1.1 (1.2)	0.9 (1.0)	0.13
Locality			
Urban	3 420 537 (93.2)	2 225 473 (96.8)	0.17
Rural	250 827 (6.8)	74 145 (3.2)	0.17
Census region			
Northeast	857 817 (23.4)	482 594 (21.%)	0.06
Midwest	724 815 (19.7)	384 138 (16.7)	0.08
South	1 379 270 (37.6)	984 238 (42.8)	0.11
West	709 462 (19.3)	448 648 (19.5)	<0.01
Area Deprivation Index, mean (SD)	66.1 (18.0)	65.2 (18.6)	0.05
Plan type			
HMO	NA	1 274 708 (55.4)	NA
PPO	NA	1 024 910 (44.6)	NA
Medicare Advantage parent insurer			
UnitedHealth	NA	833 951 (36.3)	NA
Humana	NA	491 848 (21.4)	NA
CVS	NA	220 000 (9.6)	NA
Cigna	NA	50 249 (2.2)	NA
BCBS Association	NA	150 140 (6.5)	NA
Centene	NA	36 327 (1.6)	NA
Anthem	NA	9938 (0.4)	NA
All others	NA	507 165 (22.1)	NA

Abbreviations: BCBS, Blue Cross Blue Shield; HMO, health maintenance organization; LIS, low-income subsidy; NA, not applicable; PPO, preferred provider organization; SMD, standardized mean difference.

^a^ The study sample included beneficiaries aged 65 years and older residing in the US with complete demographic information who were enrolled in Parts A, B, and D for all months alive during the year among traditional Medicare beneficiaries and Part C with Part D coverage for Medicare Advantage beneficiaries. We excluded beneficiaries who switched between traditional Medicare and Medicare Advantage at any point midyear, thus limiting the sample to those continuously enrolled in either Medicare Advantage or traditional Medicare. Using a previously validated approach, we excluded Medicare Advantage beneficiaries who were not in highly reliable and complete Medicare Advantage contracts.

^b^ Other race and ethnicity was defined as American Indian or Alaska Native, Asian or Pacific Islander, and any race or ethnicity not otherwise specified.

Compared with TM beneficiaries, MA beneficiaries who were included in the sample were similar with regard to age, sex, LIS status, dual eligibility status, and ADI (SMDs < 0.10) (Table 1). TM beneficiaries were more likely than MA beneficiaries to be White (3 025 837 beneficiaries [82.4%] vs 1 668 506 beneficiaries [72.6%]; SMD = 0.24) or to live in a rural area (250 827 beneficiaries [6.8%] vs 74 145 beneficiaries [3.2%]; SMD = 0.17) and were less likely to be from the South (1 379 270 beneficiaries [37.6%] vs 984 238 beneficiaries [42.8%]; SMD = 0.11). The 7 largest MA insurers enrolled 1 792 453 MA beneficiaries (77.9%) in the study sample, of which UnitedHealth (833 951 beneficiaries [36.3%]), Humana (491 848 beneficiaries [21.4%]), and CVS (220 000 beneficiaries [9.6%]) had the largest share. Differences in beneficiary characteristics across major MA insurers are listed in eTable 2 in Supplement 1.

### Differences in LVS Utilization Between MA and TM

Overall, the adjusted total LVS utilization across all categories was lower among MA beneficiaries compared with those enrolled in TM (50.02 vs 52.48 services per 100 beneficiary-years; adjusted absolute difference, −2.46 services per 100 beneficiary-years; 95% CI, −3.16 to −1.75 services per 100 beneficiary-years; *P* < .001) (Table 2). Differences were mainly owing to lower use of common medical treatments (eg, antibiotics for acute upper respiratory and ear infections), cardiovascular testing, procedures and surgeries, preoperative evaluation, and cancer screening (eTable 3 in Supplement 1). Utilization of low-value diagnostic and preventive testing was slightly more common among MA beneficiaries compared with TM (9.03 vs 6.47 services per 100 beneficiary-years; adjusted absolute difference, 2.56 services per 100 beneficiary-years; 95% CI, 2.11 to 3.01 services per 100 beneficiary-years; *P* < .001), as was utilization of low-value imaging services (16.67 vs 16.04 services per 100 beneficiary-years; adjusted absolute difference, 0.63 services per 100 beneficiary-years; 95% CI, 0.14 to 1.12 services per 100 beneficiary-years; *P* = .01).

**Table 2.** Adjusted Rates of LVS per 100 Beneficiary-Years in TM vs MA^a^

LVS	Rate per 100 beneficiary-years		Adjusted absolute difference per 100 beneficiary-years (95% CI)	Relative adjusted difference (95% CI), %	*P* value
	MA	TM			
Total LVS among eligible sample	50.02	52.48	−2.46 (−3.16 to −1.75)	−4.68 (−5.99 to −3.36)	<.001
Common treatments	58.13	75.71	−17.58 (−19.31 to −15.85)	−23.22 (−25.29 to −21.08)	<.001
Imaging	16.67	16.04	0.63 (0.14 to 1.12)	3.94 (0.89 to 7.08)	.01
Diagnostic and preventative testing	9.03	6.47	2.56 (2.11 to 3.01)	39.58 (31.76 to 47.86)	<.001
Cardiovascular testing	8.49	9.35	−0.87 (−1.09 to −0.64)	−9.27 (−11.53 to −6.95)	<.001
Procedures and surgeries	1.21	1.37	−0.16 (−0.24 to −0.08)	−11.72 (−17.05 to −6.05)	<.001
Preoperative evaluation	8.86	9.62	−0.76 (−0.89 to −0.64)	−7.90 (−9.16 to −6.63)	<.001
Cancer screening	13.38	13.88	−0.50 (−0.78 to −0.21)	−3.58 (−5.58 to −1.54)	<.001

Abbreviations: LVS, low-value services; MA, Medicare Advantage; TM, traditional Medicare.

^a^ Overdispersed Poisson regression models were used and adjusted for age, sex, race and ethnicity, dual eligibility status, and Centers for Medicare & Medicaid Services hierarchical condition category risk score (with an 11% deflation of hierarchical condition category risk score among MA beneficiaries). Models also included hospital referral region fixed-effects and offsets for beneficiaries who died during the year.

Within MA, use of LVS was lower among beneficiaries enrolled in HMOs compared with those enrolled in PPOs (48.03 vs 52.66 services per 100 beneficiary-years; adjusted absolute difference, −4.63 services per 100 beneficiary-years; 95% CI, −5.53 to −3.74 services per 100 beneficiary-years; *P* < .001) (Table 3). These differences were primarily owing to differences in common medical treatments, imaging, preoperative evaluation, and cancer screening services (for full list by specific LVS, see eTable 4 in Supplement 1).

**Table 3.** Adjusted Rates per 100 Beneficiary-Years Among Medicare Advantage Beneficiaries Enrolled in HMOs vs PPOs^a^

Low-value services	Rate per 100 beneficiary-years		Adjusted absolute difference per 100 beneficiary-years (95% CI)	Relative adjusted difference (95% CI), %	*P* value
	HMO	PPO			
Total low-value services among eligible sample	48.03	52.66	−4.63 (−5.53 to −3.74)	−8.80 (−10.41 to −7.16)	<.001
Common treatments	53.82	58.74	−4.92 (−6.97 to −2.87)	−8.38 (−11.65 to −4.99)	<.001
Imaging	15.39	17.56	−2.17 (−2.69 to −1.64)	−12.35 (−15.12 to −9.50)	<.001
Diagnostic and preventative testing	9.73	8.85	0.88 (0.46 to 1.30)	9.93 (5.01 to 15.08)	<.001
Cardiovascular testing	8.57	8.66	−0.08 (−0.33 to 0.16)	−0.98 (−3.73 to 1.84)	.49
Procedures and surgeries	1.04	1.12	−0.08 (−0.24 to 0.08)	−7.00 (−19.95 to 8.05)	.34
Preoperative evaluation	8.57	9.10	−0.52 (−0.71 to −0.34)	−5.77 (−7.68 to −3.82)	<.001
Cancer screening	13.62	14.89	−1.28 (−1.65 to −0.90)	−8.57 (−10.92 to −6.16)	<.001

Abbreviations: HMO, health maintenance organization; PPO, preferred provider organization.

^a^ Overdispersed Poisson regression models were used and adjusted for age, sex, race and ethnicity, dual eligibility status, and Centers for Medicare & Medicaid Services hierarchical condition category risk score. Models also included hospital referral region fixed-effects and offsets for beneficiaries who died during the year.

### Differences by Major MA Insurer Compared With TM

The total LVS utilization among the eligible sample differed by major MA insurer group (Figure). MA beneficiaries enrolled in UnitedHealth plans (adjusted absolute difference, −2.28 services per 100 beneficiary-years; 95% CI, −3.13 to −1.42 services per 100 beneficiary-years; *P* < .001), Humana plans (adjusted absolute difference, −3.86 services per 100 beneficiary-years; 95% CI, −4.87 to −2.85 services per 100 beneficiary-years; *P* < .001), Centene plans (adjusted absolute difference −8.11 services per 100 beneficiary-years; 95% CI, −11.53 to −4.69 services per 100 beneficiary-years; *P* < .001), and plans offered by other smaller MA insurers (adjusted absolute difference −3.36 services per 100 beneficiary-years; 95% CI −4.78 to −1.94 services per 100 beneficiary-years; *P* < .001) had lower LVS utilization compared with those in TM. However, there were no significant differences in LVS among MA beneficiaries enrolled in CVS contracts (adjusted absolute difference 0.33 services per 100 beneficiary-years; 95% CI −0.80 to 1.47 services per 100 beneficiary-years; *P* = .56), Cigna (adjusted absolute difference, 2.15 services per 100 beneficiary-years; 95% CI, −9.73 to 14.03 services per 100 beneficiary-years; *P* = .72), or Anthem (adjusted absolute difference, 9.22 services per 100 beneficiary-years; 95% CI, −2.84 to 21.28 services per 100 beneficiary-years; *P* = .13). MA beneficiaries enrolled in BCBS Association plans had higher LVS use than the TM beneficiaries (adjusted absolute difference, 1.82 services per 100 beneficiary-years; 95% CI, 0.44 to 3.20 services per 100 beneficiary-years; *P* < .01).

**Figure.**
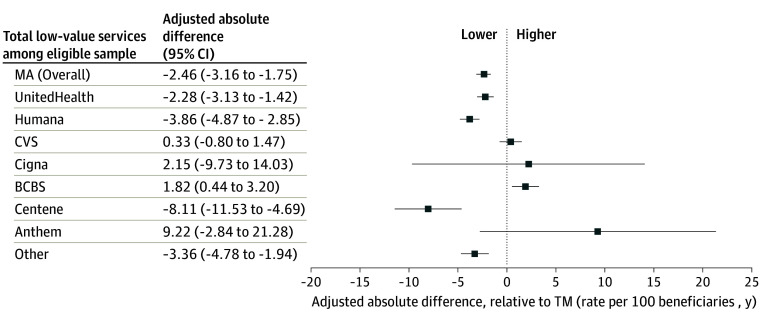
Adjusted Differences in Low-Value Services Utilization per 100 Beneficiary-Years in Traditional Medicare (TM) vs Medicare Advantage (MA) by Major Insurer BCBS indicates Blue Cross Blue Shield.

Differences in LVS utilization compared with TM both varied by insurer and by specific LVS category (eFigure in Supplement 1). In comparison with TM, all MA insurer groups had significantly lower LVS utilization for common treatments but higher utilization for diagnostic and preventative testing, although the magnitude of these differences varied substantially by MA insurer. However, for other categories, the direction and significance of differences were not consistent across MA insurer groups. For example, for imaging services, while Centene had lower utilization compared with TM (adjusted absolute difference, −1.79 services per 100 beneficiary-years; 95% CI, −3.35 to −0.22 services per 100 beneficiary-years; *P* = .03), CVS had higher utilization (adjusted absolute difference, 2.90 services per 100 beneficiary-years; 95% CI, 1.87 to 3.94 services per 100 beneficiary-years; *P* < .001), and Cigna did not have significantly different utilization compared with TM (adjusted absolute difference 1.58 services per 100 beneficiary-years; 95% CI, −4.57 to 7.73 services per 100 beneficiary-years; *P* = .61).

### Sensitivity Analyses

Unadjusted and additional sensitivity analyses applying 6% and 16% reductions to MA HCC risk scores produced similar results compared with our main models (eTable 5 in Supplement 1). In a sensitivity analysis that included beneficiaries who switched between TM and MA midyear, results were similar to our main results, with overall LVS use being lower among beneficiaries who started the year in MA, except for 1 service (cardiovascular testing), for which we no longer found a significant difference between the 2 groups (eTable 6 in Supplement 1). When comparing LVS use between MA beneficiaries enrolled in included vs excluded MA contracts, total LVS use was significantly lower among beneficiaries enrolled in excluded contracts, as can be expected given concerns that these contracts have incomplete encounter data (eTable 7 in Supplement 1). When we excluded decedents from our sample, comparing LVS use between MA and TM beneficiaries who survived the year, results were largely consistent with our primary models (eTable 8 in Supplement 1). When analyzing LVS use among BCBS enrollees in the 5 states with the highest enrollment in highly reliable and complete BCBS plans—Michigan, Alabama, North Carolina, Arizona, and Rhode Island—in comparison with TM beneficiaries in each of these states, results were heterogeneous across states (eTable 9 in Supplement 1). In Michigan, overall LVS use did not differ significantly between BCBS and TM beneficiaries; in Arizona, BCBS beneficiaries had lower LVS use, while BCBS beneficiaries in Alabama, South Carolina, and Rhode Island had higher LVS use than TM beneficiaries. When we repeated our main models using county fixed-effects instead of HRR fixed-effects, results were similar for the overall categories of LVS, with the exceptions of cardiovascular testing, which was higher in MA, and procedures and surgeries, for which there was no significant difference when using county fixed-effects (eTable 10 in Supplement 1).

## Discussion

In this national cross-sectional study of nearly 6 million Medicare beneficiaries, utilization of LVS was overall lower among those enrolled in MA compared with those enrolled in TM. Among MA beneficiaries, use of LVS was lower among those enrolled in HMOs compared with PPOs. We also found important variations in the use of LVS across the largest major MA insurer organizations. More specifically, we observed no differences in the use of LVS delivered to beneficiaries enrolled by 3 of the 7 largest MA insurer organizations in our sample compared with those enrolled in TM (including CVS, Cigna, and Anthem), and in the case of 1 large MA parent insurer organization (the BCBS Association of plans), we observed slightly higher use of LVS compared with TM.

Our study adds new evidence and nuance to existing comparisons of low-value care among beneficiaries enrolled in MA vs TM at a time when the MA program is experiencing rapid growth. While our overall results support the possibility that beneficiaries in MA may be more protected from receiving low-value care, these findings were not consistent across all major MA insurer organizations. Therefore, this study builds on prior research from Boudreau et al^11^ that focused on data from a single MA insurer and expands to a broader, more heterogeneous MA population. Additionally, this study’s sample of nearly 2.3 million MA beneficiaries builds on a survey study^10^ of approximately 5000 community-dwelling MA beneficiaries enrolled in a limited number of MA plans from 2006 to 2015 that found no significant differences in 10 of 11 LVS between MA and TM. Our study estimates higher rates of LVS use in MA and TM than those reported in the prior study by Boudreau et al.^11^ This difference may be explained in part by the inclusion of a much larger set of services in our study, differences in accounting for potential upcoding in MA, differences in the exclusion criteria for the LVS measures, and the inclusion of complex patient populations in our sample—including dual-eligible beneficiaries, patients with end-stage renal disease, and patients living in institutional settings—who generally experience higher rates of LVS at baseline.

Several possible explanations may account for lower utilization of LVS among MA beneficiaries compared with TM beneficiaries. First, MA insurers are likely acting more aggressively to manage utilization directly with tools such as prior authorizations^29^ and coverage denial.^30^ For instance, while beneficiaries in TM are rarely required to receive prior authorization (other than for a limited set of services),^31^ 99% of beneficiaries in MA are in a plan that requires prior authorizations for specific health care services.^13^ In 2022, more than 46 million prior authorization requests were submitted to MA insurers.^12^ Although greater use of prior authorizations may negatively affect care or beneficiary experience in other ways,^32^ it may contribute to lower use of LVS. Some of the results for specific LVS services may support the idea that prior authorization requirements and coverage restrictions are key mechanisms mediating the association of type of Medicare enrollment with LVS utilization. For example, vitamin D deficiency screening is one service for which TM had lower utilization than MA, which is unsurprising given that TM does not cover routine vitamin D screenings.^33^

Additional differences between MA and TM may be underlying these differential patterns of LVS utilization. For example, given the strong payment incentives to improve efficiency inherent in MA, MA insurers may be more motivated to restrict clinician networks and only include clinicians who on average prescribe and order fewer low-value medications, tests, and imaging. Moreover, value-based contracts between insurers and health care organizations, such as accountable care organizations, have been associated with small reductions in LVS utilization,^34^ likely due to greater financial alignment and possibly increased resource allocation to preventing LVS. Because uptake of risk-based alternative payment models (eg, shared savings, 2-sided risk, bundled payments, and capitated population-based payment models) has thus far been greater among MA insurers compared with TM,^35^ more beneficiaries in MA may benefit from value-based contracts and therefore receive fewer LVS.

In addition, this study is unique in its ability to examine differences in utilization of LVS across different MA insurer organizations. One possible explanation for the observed heterogeneity is that certain MA plans may be more effective in implementing the previously described utilization management strategies, such as narrow clinician networks, prior authorization, and coverage denial. A second potential explanation is that the MA insurer organizations with lower rates of LVS utilization may include more plans that are engaging in alternative payment models with their network of clinicians. While research in this area is sparse, a recent study by Gondi et al^36^ noted that advanced value-based payment models were associated with lower rates of acute care use among the MA population enrolled in Humana; we similarly found that Humana has lower rates of LVS compared with TM in our study. Another study by Cohen et al^37^ found that a 2-sided risk MA model was associated with reduced utilization of acute care compared with TM. A third possibility is that differences within MA in utilization of LVS are associated with plan structure (eg, HMO vs PPO). We found that beneficiaries in MA HMOs received fewer LVS than beneficiaries in MA PPOs, which may be explained in part by increased beneficiary motivation to seek care from in-network clinicians, who may be less likely to provide LVS. Differences in distribution of beneficiaries across HMO and PPO plans may underlie heterogeneity in LVS utilization among MA insurers.

These findings carry numerous policy and research implications. First, these results emphasize the benefits of shifting the national dialogue from the current frame of comparing MA vs TM as monoliths toward one that appreciates heterogeneity, particularly within MA. To date, an extensive literature has lumped all MA insurers together as one unit in producing comparisons of service utilization, quality of care, and value in MA vs TM.^9,10,38,39,40,41,42,43,44,45,46,47,48,49,50^ By contrast, this study suggests that the association of MA enrollment with reduced utilization of LVS does not persist across all major MA insurer organizations. Future research can work to fill this gap in research about MA, supplementing existing research gaps around other elements of MA.^38^ Second, this study offers an opportunity for learning about ways to reduce LVS utilization. Despite extensive nationwide campaigns and the devotion of substantial resources to reduce the delivery of LVS,^6,7^ utilization of LVS has remained prevalent in TM.^5,10^ Our results raise the possibility that reductions in LVS utilization could potentially be achieved in TM by learning from the approaches of MA insurers, whose beneficiaries have lower use of LVS. Such approaches may include expanding the reach of value-based payment models for fee-for-service beneficiaries, and further research can elucidate the mechanisms through which certain MA insurers are promoting higher-value care.

### Limitations

This study has several limitations. First, we used a specific set of LVS measures, which may not generalize broadly to all low-value care services. However, these metrics capture diverse aspects of patient care and are widely used in other settings.^5,8^ Second, we were not able to directly assess the effect of potential mediators of less LVS use (eg, network design or prior authorization) given that this information is not available in Medicare administrative claims and encounter data. Nonetheless, this study uses comparisons of MA HMOs and MA PPOs to offer an indirect assessment of the association of network design with LVS use within MA. However, given that HMOs are underrepresented among the included MA contracts with higher data reliability and completeness, it is possible that these findings may not generalize to all MA HMO plans. Additionally, if LVS utilization is indeed lower among HMOs, then it may also be the case that this study underestimates the difference in LVS use between MA and TM. Third, concerns exist regarding differences in beneficiary characteristics and risk profiles between beneficiaries in MA vs TM, and unobserved differences in beneficiary factors may remain after adjustment. It is possible that some of the differences in LVS utilization may be partially due to selection effects that are unrelated to differences in the design of the MA and TM programs. If beneficiaries who choose to enroll in MA vs those who enroll in TM systematically differ in their tendencies to utilize LVS at baseline, then differences in LVS utilization rates between MA and TM may reflect this selection bias. This study’s findings were robust to numerous sensitivity analyses, including deflation of HCC risk scores beyond levels used by policymakers. Fourth, it is important to note that this study’s findings are based on data from 2018 and therefore may not reflect more recent patterns of LVS utilization in the Medicare program. Fifth, we were limited to using data from the first 3 months of 2018 to determine appropriate clinical exclusions for our LVS measures, which explains some variation in our findings when compared with other studies. Sixth, while this study found significant differences in LVS utilization across different MA insurer organizations, these results should be interpreted with caution given that there remains substantial plan heterogeneity within any given insurer group—a notable example being the BCBS Association, which despite owning and managing the trademark, is an association of smaller, independent, locally operated companies, each of which offers multiple MA insurance products.^51^ Thus, there is likely further unexamined heterogeneity in LVS utilization rates across the plans within particular insurer groups.

## Conclusions

In this national cross-sectional study, LVS utilization was lower among MA beneficiaries compared with TM beneficiaries. However, meaningful differences existed within MA, such that beneficiaries in multiple large MA insurer organizations experienced LVS utilization that was no different or higher than LVS utilization experienced by beneficiaries in TM. This observation extends prior literature documenting lower use of LVS in MA compared with TM and underscores the need for further research studying the quality and value of care in MA vs TM.

## Notes

Supplement 1. **eAppendix 1.** Sample Selection of Medicare Enrollees **eAppendix 2.** Identification of Highly Reliable MA Contracts and Calculation of Risk Scores **eAppendix 3.** Definitions of Low-Value Care Services by Milliman MedInsight Health Waste Calculator **eTable 1.** Patient Characteristics of Included vs. Excluded Medicare Advantage Beneficiaries **eTable 2.** Patient Characteristics of Medicare Advantage Beneficiaries, Overall and by Payer **eTable 3.** Adjusted Rates Across Specific Low-Value Services per 100 Beneficiary-Years in Traditional Medicare and Medicare Advantage **eTable 4.** Adjusted Rates Across Specific Low-Value Services per 100 Beneficiary-Years Among MA Beneficiaries Enrolled in HMOs Versus PPOs **eFigure.** Adjusted Rates of LVS per 100 Beneficiary-Years in Traditional Medicare vs. Medicare Advantage by Major Payer **eTable 5.** Unadjusted and Adjusted Rates of LVS per 100 Beneficiary-Years in Traditional Medicare and Medicare Advantage **eTable 6.** Adjusted Rates of LVS per 100 Beneficiary-Years in Traditional Medicare vs. Medicare Advantage, Including Beneficiaries Who Switched During the Year **eTable 7.** Adjusted Rates of LVS per 100 Beneficiary-Years in Medicare Advantage, Comparison of Included vs Excluded MA Plans **eTable 8.** Adjusted Rates of LVS per 100 Beneficiary-Years in Traditional Medicare vs. Medicare Advantage, Excluding Beneficiaries Who Died During the Year **eTable 9.** Adjusted Rates of LVS per 100 Beneficiary-Years in BCBS Plans vs. TM **eTable 10.** Adjusted Rates of LVS per 100 Beneficiary-Years in Traditional Medicare vs. Medicare Advantage With County Fixed Effects

Supplement 2.
Data Sharing Statement
