# Supplementary material for: Evaluation of Low-Value Services Across Major Medicare Advantage Insurers and Traditional Medicare
Source: JAMA Netw Open. 2024 Nov 1;7(11):e2442633. doi: 10.1001/jamanetworkopen.2024.42633 (PMC11530944; doi:10.1001/jamanetworkopen.2024.42633)
Supplement: Supplement 1. — eAppendix 1. Sample Selection of Medicare Enrollees eAppendix 2. Identification of Highly Reliable MA Contracts and Calculation of Risk Scores eAppendix 3. Definitions of Low-Value Care Services by Milliman MedInsight Health Waste Calculator eTable 1. Patient Characteristics of Included vs. Excluded Medicare Advantage Beneficiaries eTable 2. Patient Characteristics of Medicare Advantage Beneficiaries, Overall and by Payer eTable 3. Adjusted Rates Across Specific Low-Value Services per 100 Beneficiary-Years in Traditional Medicare and Medicare Advantage eTable 4. Adjusted Rates Across Specific Low-Value Services per 100 Beneficiary-Years Among MA Beneficiaries Enrolled in HMOs Versus PPOs eFigure. Adjusted Rates of LVS per 100 Beneficiary-Years in Traditional Medicare vs. Medicare Advantage by Major Payer eTable 5. Unadjusted and Adjusted Rates of LVS per 100 Beneficiary-Years in Traditional Medicare and Medicare Advantage eTable 6. Adjusted Rates of LVS per 100 Beneficiary-Years in Traditional Medicare vs. Medicare Advantage, Including Beneficiaries Who Switched During the Year eTable 7. Adjusted Rates of LVS per 100 Beneficiary-Years in Medicare Advantage, Comparison of Included vs Excluded MA Plans eTable 8. Adjusted Rates of LVS per 100 Beneficiary-Years in Traditional Medicare vs. Medicare Advantage, Excluding Beneficiaries Who Died During the Year eTable 9. Adjusted Rates of LVS per 100 Beneficiary-Years in BCBS Plans vs. TM eTable 10. Adjusted Rates of LVS per 100 Beneficiary-Years in Traditional Medicare vs. Medicare Advantage With County Fixed Effects [file jamanetwopen-e2442633-s001.pdf]

Supplemental Online Content

Duggan C, Beckman AL, Ganguli I, et al. Evaluation of low-value services across major Medicare Advantage insurers and traditional Medicare. *JAMA Netw. Open*. 2024;7(11):e2442633. doi:10.1001/jamanetworkopen.2024.42633

eAppendix 1. Sample Selection of Medicare Enrollees
eAppendix 2. Identification of Highly Reliable MA Contracts and Calculation of Risk Scores
eAppendix 3. Definitions of Low-Value Care Services by Milliman MedInsight Health Waste Calculator
eTable 1. Patient Characteristics of Included vs. Excluded Medicare Advantage Beneficiaries
eTable 2. Patient Characteristics of Medicare Advantage Beneficiaries, Overall and by Payer
eTable 3. Adjusted Rates Across Specific Low-Value Services per 100 Beneficiary-Years in Traditional Medicare and Medicare Advantage
eTable 4. Adjusted Rates Across Specific Low-Value Services per 100 Beneficiary-Years Among MA Beneficiaries Enrolled in HMOs Versus PPOs
eFigure. Adjusted Rates of LVS per 100 Beneficiary-Years in Traditional Medicare vs. Medicare Advantage by Major Payer
eTable 5. Unadjusted and Adjusted Rates of LVS per 100 Beneficiary-Years in Traditional Medicare and Medicare Advantage
eTable 6. Adjusted Rates of LVS per 100 Beneficiary-Years in Traditional Medicare vs. Medicare Advantage, Including Beneficiaries Who Switched During the Year
eTable 7. Adjusted Rates of LVS per 100 Beneficiary-Years in Medicare Advantage, Comparison of Included vs Excluded MA Plans
eTable 8. Adjusted Rates of LVS per 100 Beneficiary-Years in Traditional Medicare vs. Medicare Advantage, Excluding Beneficiaries Who Died During the Year
eTable 9. Adjusted Rates of LVS per 100 Beneficiary-Years in BCBS Plans vs. TM
eTable 10. Adjusted Rates of LVS per 100 Beneficiary-Years in Traditional Medicare vs. Medicare Advantage With County Fixed Effects

This supplemental material has been provided by the authors to give readers additional information about their work.

© 2024 Duggan C et al. *JAMA Network Open.*

eAppendix 1. Sample Selection of Medicare Enrollees

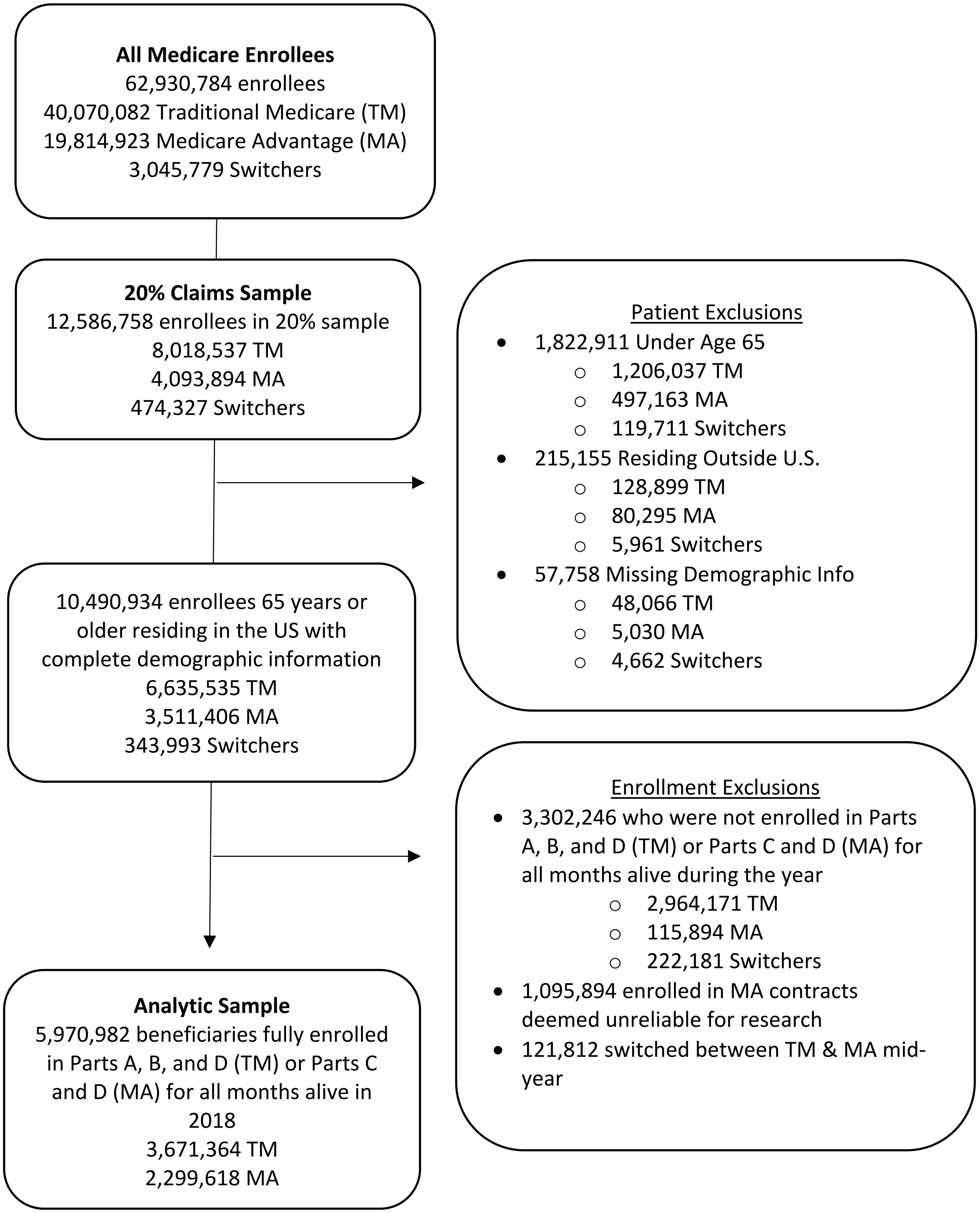

© 2024 Duggan C et al. *JAMA Network Open.*

eAppendix 2. Identification of Highly Reliable MA Contracts and Calculation of Risk Scores

Identification of highly reliable MA contracts

Prior research has noted incompleteness of MA encounter data files from before the year 2018. To address this concern, this study used a validated approach by Jung et al. 2022 to identify MA contracts with high reliability of completeness. Specifically, we used a methodology that compares MA encounter data with other external data sources (e.g., the CMS Medicare Provider Analysis and Review (MedPAR) file and the Healthcare Effectiveness Data and Information Set (HEDIS)) on values like number of ED visits, number of ambulatory care visits, and number of inpatient stays. Based on the analysis performed by Jung et al., 499 MA contracts were excluded from this study's analysis and 210 contracts in 2018 MA encounter data were included in this study's analysis. A comparison of characteristics of MA beneficiaries that were included vs. excluded is presented in Supplement Table 1.

Citations:

- Medicare Payment Advisory Commission (MedPAC). The Medicare Advantage Program: status report 2020. Available from: https://www.medpac.gov/wp-content/uploads/import_data/scrape_files/docs/default-source/reports/mar20_medpac_ch13_sec.pdf.
- Beckman AL, Frakt AB, Duggan C, Zheng J, Orav EJ, Tsai TC, et al. Evaluation of Potentially Avoidable Acute Care Utilization Among Patients Insured by Medicare Advantage vs Traditional Medicare. JAMA Health Forum. 2023;4(2):e225530. Epub 20230203. doi: 10.1001/jamahealthforum.2022.5530. PubMed PMID: 36826828; PubMed Central PMCID: PMC9958527.
- Jung J, Carlin C, Feldman R. Measuring resource use in Medicare Advantage using Encounter data. Health Serv Res. 2022;57(1):172-81. Epub 20211006. doi: 10.1111/1475-6773.13879. PubMed PMID: 34510453; PubMed Central PMCID: PMC8763275.
- Jung J, Carlin C, Feldman R, Tran L. Implementation of resource use measures in Medicare Advantage. Health Serv Res. 2022;57(4):957-62. Epub 20220411. doi: 10.1111/1475-6773.13970. PubMed PMID: 35411550.

Calculation of risk scores

As noted in the primary manuscript, in this study, we excluded chart review claims from MA encounter records in calculating HCC scores, given that the phenomenon of inflated coding may be particularly evident in these records. For similar reasons, this study limited HCC risk score calculations to the inpatient and outpatient files only for both MA and TM beneficiaries. The HCC calculation also excluded home health nursing visit and carrier file records to reduce concerns of upcoding in MA.

© 2024 Duggan C et al. *JAMA Network Open.*

eAppendix 3. Milliman MedInsight Health Waste Calculator Specifications for 35 Measures of Low-Value Care

See the table below for specifications provided by Milliman for the 35 low-value service measures we identified using the proprietary Milliman MedInsight Health Waste Calculator software program (version 8.0).

Measure ID	Name	Starting Population	Exclusion	Not Wasteful	Likely Wasteful	Wasteful	Cascading Event	Citations
**AACE04**	**Total or Free T3 Level**	Members of all age groups who obtained serum total or free T3 test and an Evaluation and Management claim that also contains a diagnosis of hypothyroidism and a prescription for levothyroxine	None	None	None	All starting population	None	https://www.liebertpub.com/doi/pdf/10.1089/thy.2012.0205
**ACC00**	**Cardiac stress testing**	All members aged 18 years and older with a service for cardiac stress testing	· All inpatient admissions, emergency care or observation care<br>· Members with a service for coronary angiography<br>· Members with a service for PCI/CABG	· Members with a service for stress testing (stress EKG, cardiac radionuclide imaging, and stress echo) and a diagnosis of cardiac symptoms or ventricular tachycardia<br>· Members with a service for stress CMR and a diagnosis of ventricular tachycardia<br>· Members with a service for	· Members with a service for stress EKG and a diagnosis of cardiac conditions<br>· Member aged more than 40 years with 2 or more different risk factors among diabetes mellitus or hypertension or hyperlipidemia or obesity or coronary artery disease or	All remaining.	Members with PCI/CABG after a wasteful or likely wasteful cardiac stress testing.	http://annals.org/aim/article/1363528/screening-coronary-heart-disease-electrocardiography-u-s-preventive-services-task

© 2024 Duggan C et al. *JAMA Network Open.*

				advanced stress testing (cardiac radionuclide imaging, stress echo or stress CMR) and a diagnosis of cardiac conditions<br>· Members with a service for stress EKG with cardiac rehabilitation and a diagnosis of heart failure<br>· Members with a service for stress echocardiography and a diagnosis of valve disease or cardiomyopathy<br>· Members with a service for kidney or liver transplant and a diagnosis of pre-operative cardiovascular examination	peripheral artery disease			
**ACOE03**	**Imaging for Diagnosis of Plantar Fasciitis**	All members aged 18 years and older who obtained X-ray imaging service with an Evaluation and Management claim that also contains a diagnosis of plantar fasciitis	All members with a diagnosis of plantar fasciitis within 1 year prior to the WC index claim	- All members with a diagnosis of calcaneal tuberosity or body fracture<br>- All members with a diagnosis of subtalar and talonavicular arthritis<br>- All members with a diagnosis of retrocalcaneal bursitis	None	All remaining.	None	https://www.jospt.org/doi/full/10.2519/jospt.2008.0302?url_ver=Z39.88-2003&rfr_id=ori:rid:crossref.org&rfr_dat=cr_pub%20%200pubmed

© 2024 Duggan C et al. *JAMA Network Open.*

**ACPY01**	**Brain Imaging Studies (CT or MRI) for Simple Syncope**	All members aged 18 years and older who obtained brain imaging studies (CT or MRI) for syncope	· All inpatient admissions<br>· Competing diagnosis for brain imaging<br>· Diagnosis of Head Injury<br>· Diagnosis of benign or malignant tumors of the head and neck	Diagnosis of neurological deficits	None	All remaining.	None	http://www.acr.org/~/media/1C1F7C7A570D469A9C411D95067BDF94.pdf
**ACR01**	**Imaging for Uncomplicated Headache**	All members aged 18 years and older with a diagnosis of uncomplicated headache who obtained a service for imaging	- Members with inpatient admissions<br>- Members with diagnosis of cancer or head trauma<br>- Members with a diagnosis of complicated sinusitis/mastoiditis/middle ear disease	- All members aged 55 years or older with a diagnosis of raised ESR or temporal arteritis without the diagnosis of headache who obtained a service for MRI/MRA Head<br>- All members with a diagnosis of complicated headache (Thunderclap/horner syndrome/vertebral dissection) who obtained a service for CT/MRI/CTA/MRA<br>- Members with with a diagnosis of underlying conditions (post traumatic headache, neurologic deficit, epilepsy, ataxia)	- All members aged 55 years or older with a diagnosis of raised ESR or temporal arteritis without the diagnosis of headache who obtained a service for CT/CTA<br>- Members with diagnosis of chronic conditions (trigeminal headache, immunocompromised) who obtained a service for CT/MRA/CTA<br>- Members with a diagnosis of underlying conditions (post-traumatic headache,	All remaining	None	https://www.aafp.org/afp/2013/0515/p682.pdf

© 2024 Duggan C et al. *JAMA Network Open.*

				who obtained a service for MRI/CT<br>- Members with a diagnosis of pregnancy without a diagnosis of headache who obtained a service for MRI/CT<br>- Members with a diagnosis of meningitis/encephalitis who obtained a service for MRI<br>- Members with a diagnosis of chronic conditions (Trigeminal headache, immunocompromised conditions) who obtained a service for MRI<br>- Members with a diagnosis of cerebrovascular event who obtained a service for MRI/MRA/CT	neurologic deficit, epilepsy, ataxia) who obtained a service for MRA/CTA<br>- Members with a diagnosis of meningitis/encephalitis who obtained a service for CT<br>- Members with a diagnosis of chronic headache who obtained a service for MRI			
**ACRH03**	**MRI for Rheumatoid Arthritis**	All members aged 18 years and older with a diagnosis of rheumatoid arthritis who obtained an MRI study	None	None	None	All starting population	None	http://ard.bmj.com/content/annrheumdis/72/6/804.full.pdf
**AFP00**	**Cervical Cancer Screening in Women**	All female members who obtained a cervical cancer	Members with a diagnosis of HIV	· Members aged 21 years and older with a diagnosis of high risk conditions	Members aged 21 years or older years and a diagnosis of	All remaining.	Colposcopy, cervical biopsy and outpatient	http://www.uspreventiveservicestaskforce.o

© 2024 Duggan C et al. *JAMA Network Open.*

		screening related service		for developing cervical cancer or with gynecologic malignancy or dysplasia<br>· Members aged 21 years or older and any documented abnormal Pap smear findings<br>· Members aged between 21 and 64 years who had cervical cytology and no codes of total hysterectomy<br>· Members aged between 30 and 64 years who had cervical cytology and HPV testing on the same day and no codes of total hysterectomy<br>- Members aged between 30 and 64 years who had HPV testing once in 5 years and no codes of total hysterectomy	potential cervical cancer risk conditions		gynecology visits are identified as cascading events if they occur after a Likely Wasteful or Wasteful cervical cancer screening.	rg/Page/Document/RecommendationStatementFinal/cervical-cancer-screening
**AFP02**	**Imaging for Low Back Pain**	All members 18 years of age and older with a diagnosis of low back pain who obtained imaging service for low back pain	· History of Low Back Pain<br>· Lumbar spine surgery<br>· Inpatient admission	· Members with diagnosis of any neurological deficits who obtained an MRI imaging for low back pain<br>· Members with other serious underlying conditions (cancer,	· Members with diagnosis of any other serious underlying conditions (cancer, immunosuppression) who obtained a service for X-ray/CT lumbar	All remaining.	Back surgeries in the absence of fractures are identified as cascading if they occur	https://acsearch.acr.org/docs/69483/Narrative/

© 2024 Duggan C et al. *JAMA Network Open.*

				immunosuppression) who obtained an MRI imaging for low back pain<br>· Members with conditions requiring imaging (osteoporosis, trauma, drug abuse, infection) who obtained an MRI imaging for low back pain<br>· Member aged 70 years and older who obtained X-ray/CT lumbar spine without contrast/MRI without contrast<br>· Members with diagnosis of any conditions requiring imaging (osteoporosis, trauma, drug abuse, infection) who obtained X-ray/CT lumbar spine without contrast<br>· Members with diagnosis of long term steroid use who obtained an imaging service for X-ray/CT lumbar spine without contrast/MRI without contrast	spine<br>· Members with diagnosis of neurological deficits who obtained a service for CT lumbar spine		after a Likely Wasteful or Wasteful back imaging service. Physical therapy after back surgery, and chiropractor or physician visits after back surgery are also considered cascading events.	

© 2024 Duggan C et al. *JAMA Network Open.*

**AFP03**	**DEXA Screening for Osteoporosis**	All women under 65 years of age and men 50-69 years of age who obtained (DEXA) screening for osteoporosis	- Diagnosis of osteoporosis<br>- Members with a prescription for High Risk Medications and days' supply of = 90 days or more	· Members with diagnosis of major risk factors for developing osteoporosis<br>· Members with at least two potential risk factors for developing osteoporosis	None	All remaining.	None	http://annals.org/aim/article/746858/screening-osteoporosis-u-s-preventive-services-task-force-recommendation-statement
**AFP05**	**EKGs and Other Cardiac Screens**	All members aged 18 years and older with a service for EKG or any other cardiac screening	- Members with other inflammatory conditions such as arthritis, joint pains, myositis etc.<br>- Members who obtained preoperative cardiac screening tests<br>- Members who had low risk surgery<br>- Members with an inpatient stay	- Members with diagnosis of high risk markers for CHD<br>- Members with diagnosis of two or more risk factors suggestive of intermediate CHD<br>- Members with two or more signs or symptoms suggestive of CHD	None	All remaining	CABG/PCI and outpatient cardiology visit after a wasteful or likely wasteful EKG is identified as cascading events.	https://www.uspreventiveservicestaskforce.org/Page/Document/RecommendationStatementFinal/coronary-heart-disease-screening-with-electrocardiography
**AI02**	**Immunoglobulin G (IgG) or Immunoglobulin E (IgE) Tests in the Evaluation of Allergy**	All members with a diagnosis of allergy who obtained IgG or IgE testing	None	· Members with a diagnosis of eczema or dermatographism who had IgE allergy test<br>· Children less than 15 years old who had IgE allergy test	· Members with a diagnosis of migraine and food allergy who had IgG allergy test<br>· Members with a diagnosis of atopic allergy who had IgE allergy test	All remaining.	None	http://www.aaaai.org/Aaaai/media/MediaLibrary/PDF%20Documents/Practice%20and%20Parameters/allerg

© 2024 Duggan C et al. *JAMA Network Open.*

								ydiagnostic testing.pdf
**AI03**	**Diagnostic Testing for Chronic Urticaria**	All members who had routine diagnostic testing for more than once for chronic urticaria.	Members with not more than one allergy diagnostic test service or where the count of the allergy test is more than one but without a diagnosis of urticaria.	None	None	All remaining.	None	https://www.aaaai.org/Aaaai/media/MediaLibrary/PDF%20Documents/Practice%20and%20Parameters/Urticaria-2014.pdf
**AN01**	**Electroencephalography for Headaches**	All members with a diagnosis of headache who obtained EEG	All inpatient admission	· Diagnosis of epilepsy or seizures or Sleep Disorders<br>· Diagnosis of abnormal involuntary movement	None	All remaining.	None	http://staywell.com/wp-content/uploads/2013/12/Headache0113.pdf
**AN02**	**Imaging of the Carotid Arteries**	All members aged 18 years and older who obtained a carotid duplex ultrasound imaging	All inpatient admission	Members with neurological deficits	None	All remaining.	None	https://www.ncbi.nlm.nih.gov/pmc/articles/PMC3295536/

© 2024 Duggan C et al. *JAMA Network Open.*

**AO02**	**Imaging Tests for Eye Disease**	All members who obtained an eye imaging test	None	· Members with conditions requiring posterior optical coherence tomography with ophthalmologist visit or office visit with ophthalmologist or optometrist specialty who obtained posterior optical coherence tomography<br>· Members with conditions requiring anterior optical coherence tomography with ophthalmologist visit or office visit with ophthalmologist or optometrist specialty who obtained anterior optical coherence tomography<br>· Members with conditions requiring fundus photography with ophthalmologist visit or office visit with ophthalmologist or optometrist specialty who obtained fundus photography<br>· Members with conditions requiring	None	All remaining.	None	https://www.aao.org/preferred-practice-pattern/comprehensive-adult-medical-eye-evaluation-2015

© 2024 Duggan C et al. *JAMA Network Open.*

				visual field testing with ophthalmologist visit or office visit with ophthalmologist or optometrist specialty who obtained visual field testing<br>· Members with conditions requiring external eye photography with ophthalmologist visit or office visit with ophthalmologist or optometrist specialty who obtained external eye photography<br>· Members with conditions requiring internal eye photography with ophthalmologist visit or office visit with ophthalmologist or optometrist specialty who obtained internal eye photography				

© 2024 Duggan C et al. *JAMA Network Open.*

**AOHN01**	**CT Head/Brain for Sudden Onset Hearing Loss**	All members with a diagnosis of sudden onset hearing loss who obtained a CT scan of the head/brain	Members with any competing diagnosis	· Members with a diagnosis of acoustic neuroma or cerebrovascular accident who can not receive MRI scanning<br>· Pregnancy<br>· Members with a diagnosis of history of trauma involving the ear<br>· Members with a history of chronic ear disease or other diseases (Paget disease, fibrous dysplasia, encephalopathy or bone metastasis to the temporal bone, benign or malignant tumors of the petrous temporal bone, etc.)	None	All remaining.	None	http://journals.sagepub.com/doi/pdf/10.1177/0194599812436449
**AOHN04**	**Imaging for Uncomplicated Acute Rhinosinusitis**	All members with a diagnosis of uncomplicated acute rhinosinusitis who obtained sinus imaging	- Members with Inpatient admissions<br>- Members with competing diagnosis	- Members who obtained sinus / head CT and have a diagnosis of chronic sinusitis or with any diagnosis of acute or recurrent sinusitis<br>- Members with a diagnosis of complicated rhinosinusitis (orbital or intracranial complications with ocular or neurologic	Members a diagnosis of immunodeficiency or acute frontal or sphenoidal sinusitis who obtained sinus CT	All remaining.	Sinus surgery without diagnosis for chronic sinusitis, recurrent sinusitis or complicated rhinosinusitis are identified as cascading events if	https://www.aafp.org/afp/2016/0715/p97.pdf

© 2024 Duggan C et al. *JAMA Network Open.*

				deficits, preseptal or post septal cellulitis, sub periosteal abscess, orbital cellulitis or abscess, cavernous sinus thrombosis, osteomyelitis of frontal bone, subdural empyema, epidural or brain abscess, meningitis, brain infarction or myotic aneurysm, sinonasal obstruction, suspected mass lesion) who obtained sinus CT/MRI			they occur after a Likely Wasteful or Wasteful sinus imaging.	
**AP00**	**Antibiotics for acute upper respiratory and ear infections**	All members aged 3 months and older with a diagnosis of URI or ear infection (acute sinusitis, URI, viral respiratory illness or acute otitis externa) who were prescribed oral antibiotics	· Members with any diagnosis for comorbid conditions<br>· Members with any competing diagnosis<br>· Members with a service for tympanostomy tube placement<br>· Members with a diagnosis of otitis media	· Members with a diagnosis for acute rhinosinusitis and a diagnosis of sinusitis complications<br>· Members with a diagnosis of acute otitis externa and a diagnosis of middle ear disease<br>· Members with a diagnosis for malignant otitis externa	None	All remaining.	Members with likely wasteful and wasteful services with clostridium difficile infection, allergy due to antibiotics infectious diarrhea, allergy due to antibiotics, GI disturbanc e and	http://annals.org/aim/article/2481815/appropriate-antibiotic-use-acute-respiratory-tract-infection-adults-advice-high

© 2024 Duggan C et al. *JAMA Network Open.*

							antibiotic resistance.	
**APA01**	**Concurrent Use of Two or More Antipsychotic Medications**	All members who were prescribed antipsychotics	Prescription for lithium within 60 days prior to or on the trigger event.	Members without any concurrent prescription of 2 different antipsychotic medication within 30 days consecutively for a period of 60 days prior to or on the trigger event.	None	All remaining	Adverse effects associated with antipsychotic polypharmacy with an ER admission are identified as cascading events if they occur after a Likely Wasteful or Wasteful prescriptions of concurrent antipsychotic medications.	http://www.choosingwisely.org/clinician-lists/american-psychiatric-association-routine-prescription-of-two-or-more-concurrent-antipsychotics/
**ASA01a**	**Preoperative Baseline Laboratory Studies**	All members aged 2 years or older who obtained a baseline laboratory testing prior to an elective low risk surgery.	· E&M visit for emergency care, observation or urgent care<br>· Members who obtained electrolyte testing laboratory related services and prescription of medications	Members who obtained urinalysis prior to urologic procedure or after the diagnosis of urinary symptoms or disorders	None	All remaining.	None	http://anesthesiology.pubs.asahq.org/article.aspx?articleid=2443414

© 2024 Duggan C et al. *JAMA Network Open.*

			such as digoxin, diuretics and angiotensin converting enzyme inhibitors or angiotensin receptor blockers<br>· Members with a diagnosis of endocrine, liver or renal disorders<br>· Members who obtained CBC testing related services and a history of anemia or history suggestive of recent blood loss<br>· Members who obtained coagulation testing related services and a diagnosis of coagulation disorders or a prescription of anticoagulant medications						

© 2024 Duggan C et al. *JAMA Network Open.*

**ASA01b**	**Preoperative EKG, Chest X-Ray and Pulmonary Function Testing**	All members aged 2 years or older who obtained an EKG, chest X-ray and pulmonary function testing prior to an elective low risk surgery.	E&M visit for emergency care, observation or urgent care	· Members with cardiovascular risk factors<br>· Members with signs and symptoms of cardiovascular and cardiopulmonary disease<br>· Members with a diagnosis of underlying pulmonary disease	None	All remaining.	Members with PCI/CABG after a wasteful or likely wasteful pre-operative EKG.	http://anesthesiology.pubs.asahq.org/article.aspx?articleid=2443414
**ASA02**	**Preoperative Cardiac Echocardiography or Stress Testing**	All members aged 18 years or older who obtained an echocardiography or stress testing prior to a low or intermediate risk non-cardiac surgery	· Inpatient admission<br>· E&M visit for emergency care, observation or urgent care<br>· Members with diagnosis of high risk markers for CHD	Members with diagnosis of two or more different signs or symptoms suggestive of CHD	None	All remaining.	Members with PCI/CABG after a wasteful or likely wasteful pre-operative echocardiography or stress testing.	http://circ.ahajournals.org/content/130/24/2215.long
**ASRO04**	**Proton Beam Therapy for Prostate Cancer**	All male members 18 years and older with a diagnosis of prostate cancer who had a service for proton beam therapy	None	All members with encounter for clinical research	None	All remaining	None	https://www.sciencedirect.com/science/article/pii/S2405632418300441

© 2024 Duggan C et al. *JAMA Network Open.*

**DOR121**	**Vertebroplasty**	All members aged 18 years and older with a service for vertebroplasty	None	Members with a diagnosis of spinal or vertebral conditions (hemangioma of spine, multiple myeloma, eosinophilic granuloma or Kummell Disease)	None	All remaining.	Members with vertebral fractures after the wasteful or likely wasteful vertebroplasty and an E&M visit or spine imaging or physical therapy associated with vertebral fracture are identified as cascading events. Pulmonary embolism and CT chest for the embolism are also identified as cascading events.	http://onlinelibrary.wiley.com/doi/10.1002/14651858.CD006349.pub2/epdf

© 2024 Duggan C et al. *JAMA Network Open.*

**DOR124**	**Renal Artery Revascularization**	All members who obtained a service for renal artery revascularization	None	Members with a diagnosis of fibromuscular dysplasia	· Members with a diagnosis of malignant hypertension<br>· Members with a diagnosis of chronic kidney disease stage III to stage VI<br>· Members with a diagnosis of pulmonary edema or acute coronary syndrome	All remaining.	Members with post-procedureal bleeding, post procedural infection, femoral artery pseudoaneurysm or dissection or disseminated thrombi and renal artery or kidney perforation after after wasteful or or likely wasteful renal artery revascularization.	http://www.nejm.org/doi/full/10.1056/NEJMoa0905368
**DOR21**	**Arthroscopic Lavage and Debridement for Knee Osteoarthritis**	All members aged 18 years and older with a diagnosis of knee osteoarthritis who obtained an arthroscopic lavage or debridement	None	None	None	All starting population	None	http://www.aaos.org/research/guidelines/TreatmentofOsteoarthritisoftheKneeGuideline.pdf
**DOR85**	**Antidepressants Monotherapy in Bipolar Disorder**	All members with a diagnosis of bipolar disorder who were prescribed antidepressants	None	Members with a prescription for mood stabilizers	None	All remaining.	None	https://www.healthquality.va.gov/bipolar/bd_306_sum.pdf

© 2024 Duggan C et al. *JAMA Network Open.*

**GE01**	**Colorectal Cancer Screening in Adults 45 Years and Older**	All members aged 45 years and older who obtained a colorectal cancer screening	· Members with a diagnosis of colorectal cancer, family or personal history of colorectal cancer or colon adenoma<br>· Members with a service for total colectomy	All members between 45-75 years of age with:<br>· FOBT once in a year; or<br>· Immunochemical-based fecal occult blood testing once in a year; or<br>· FIT-DNA once in a year; or<br>· Flexible sigmoidoscopy once in 5 years; or<br>· CT colonography once in 5 years; or<br>· Colonoscopy once in 10 years.	Members aged between 76-85 years with colorectal cancer screening services.	All remaining.	Perforation is identified as a cascading event if they occur after a Likely Wasteful or Wasteful colorectal cancer screening.	http://www.cancer.org/acs/groups/cid/documents/webcontent/003170-pdf.pdf
**JAMA06**	**PTH for CKD**	All members with a diagnosis of Chronic Kidney Disease (CKD) stage 1-3 and who had PTH testing	All members with a dialysis service	All members with a diagnosis of hypercalcemia	None	All starting population	None	https://www.ncbi.nlm.nih.gov/books/NBK328150/
**JH001**	**CT Scans for Emergency Room Evaluation of Dizziness**	All aged 18 years and older with a diagnosis of dizziness who obtained a CT scan	Members with comorbid conditions Members with Inpatient admission	All members with a diagnosis of competing diagnosis or benign or malignant tumors of the head and neck	None	All remaining.	None	https://www.ncbi.nlm.nih.gov/pmc/articles/PMC2676794/pdf/nihms102245.pdf
**SCCT01**	**Coronary Artery Calcium Scoring for Known CAD**	All members aged 18 years and older with a prior diagnosis of CAD (including stents and bypass grafts) who have had coronary	None	None	None	All starting population	None	http://circ.ahajournals.org/content/129/25_suppl_2/S49.long

© 2024 Duggan C et al. *JAMA Network Open.*

		artery calcium scoring service						
**SCP01**	**Screening for Vitamin D Deficiency**	All members who obtained a vitamin-D testing	None	· Members with a diagnosis of chronic conditions who obtained 25-OH-vitamin D testing<br>· Members with a diagnosis of risk factors who obtained 25-OH-vitamin D testing<br>· Members with a prescription for high risk medications who obtained 25-OH-vitamin D testing<br>· Members with pregnancy and obesity who obtained 25-OH-vitamin D testing<br>· Members aged 65 years or older with any history of falls or a history of non-traumatic fractures who obtained 25-OH-vitamin D testing<br>· Members with inherited or acquired disorders of vitamin D and phosphate metabolism who obtained 1,25 (OH)2 -vitamin D testing	None	All remaining.	None	http://annals.org/aim/fullarticle/1938935/screening-vitamin-d-deficiency-adults-u-s-preventive-services-task

© 2024 Duggan C et al. *JAMA Network Open.*

**SNUC01**	**Coronary Angiography**	All members aged 18 years or older who obtained a service for coronary angiography	Members with a diagnosis of cardiac transplant status or congenital cardiac anomalies	· Members with cardiac conditions (acute coronary syndrome, myocardial infarction, heart failure, or ventricular fibrillation or ventricular tachycardia)<br>· Members who obtained a service for cardiac valve surgeries<br>· Members with known obstructive CAD or prior PCI/CABG and symptoms of chronic heart disease or abnormal cardiovascular study results<br>· Members who obtained a service for stress test and symptoms of chronic heart disease and abnormal cardiovascular study results	· Members with a diagnosis of other cardiac conditions (atrial fibrillation, heart block, left bundle branch block, valve disease, cardiomyopathies, pericardial disease and stenosis/ regurgitation)<br>· Members with a diagnosis of pre-operative cardiovascular examination and 3 or more different high risk conditions (ischemic heart disease, diabetes mellitus, renal insufficiency, CVA)<br>· Members who obtained a service for stress test and symptoms of chronic heart disease or abnormal cardiovascular study results	All remaining.	Members with adverse effects of angiography, heparin induced thrombocytopenia, contrast induced nephropathy, complications of coronary angiography, myocardial infarction or respiratory insufficiency after a wasteful or likely wasteful coronary angiography.	http://www.sciencedirect.com/science/article/pii/S0735109713061470?via%3Dihub

© 2024 Duggan C et al. *JAMA Network Open.*

**STHS05**	**Pulmonary Function Testing Before Cardiac Surgery**	All members aged 18 years and older who obtained pulmonary function testing 30 days before cardiac surgery	None	- Members with diagnosis of any underlying pulmonary disease<br>- Members with diagnosis of respiratory symptoms	None	All remaining.	None	https://jamanetwork.com/journals/jama/fullarticle/2510916
**URG01**	**Prostate-Specific Antigen (PSA) Screening for Prostate Cancer**	All men who obtained a PSA-based screening test for prostate cancer	None	- Members with a diagnosis of prostate cancer or who have a risk of recurrence of prostate cancer<br>- All male members with age between 55-69 years as on the index claim	Members who have risk factors for prostate cancer	All remaining.	None	http://annals.org/aim/article/1216568/screening-prostate-cancer-u-s-preventive-services-task-force-recommendation

© 2024 Duggan C et al. *JAMA Network Open.*

e Table 1. Patient Characteristics of Included vs. Excluded* Medicare Advantage Beneficiaries

	Included MA Beneficiaries (n = 2,299,618)	Excluded MA Beneficiaries (n = 1,211,788)	SMD
**Age, Mean (SD)**	75.3 (7.3)	75.7 (7.5)	0.05
**Age Group**			
65-74	1,220,706 (53.1%)	625,264 (51.6%)	0.03
75-84	782,579 (34.0%)	412,378 (34.0%)	0.00
85+	296,333 (12.9%)	174,200 (14.4%)	0.04
**Sex, N (%)**			
Female	983,592 (42.8%)	528,002 (43.6%)	0.02
Male	1,316,026 (57.2%)	683,840 (56.4%)	0.02
**Race/Ethnicity, N (%)**			
Non-Hispanic Asian	75,546 (3.3%)	66,663 (5.5%)	0.11
Non-Hispanic Black	279,271 (12.1%)	104,717 (8.6%)	0.11
Hispanic	225,273 (9.8%)	149,048 (12.3%)	0.08
Non-Hispanic White	1,668,506 (72.6%)	857,452 (70.8%)	0.04
Other/Unknown	51,022 (2.2%)	33,962 (2.8%)	0.04
**Dual Eligibility, N (%)**			
Medicare & Medicaid Eligible	307,261 (13.4%)	210,687 (17.4%)	0.11
Medicare Only	1,992,357 (86.6%)	1,001,155 (82.6%)	0.11
**Low Income Subsidy Status, N (%)**			
Non-LIS Recipient	1,909,317 (83.0%)	964,912 (79.6%)	0.09
Partial LIS Recipient	368,287 (16.0%)	239,098 (19.7%)	0.10
Full LIS Recipient	22,014 (1.0%)	7,832 (0.6%)	0.03
**HCC Risk Score, Mean (SD)**	0.92 (1.0)	0.88 (1.0)	0.04
**Locality, N (%)**			
Urban	2,225,473 (96.8%)	1,174,556 (97.3%)	0.03
Rural	74,145 (3.2%)	32,627 (2.7%)	0.03
**Census Region, N (%)**			
Northeast	482,594 (21.0%)	290,840 (24.1%)	0.07
Midwest	384,138 (16.7%)	252,040 (20.9%)	0.11
South	984,238 (42.8%)	244,520 (20.3%)	0.50
West	448,648 (19.5%)	419,809 (34.8%)	0.35
**Area Deprivation Index, Mean (SD)**	65.2 (18.6)	61.81 (17.9)	0.18
**Plan Type, N (%)**			
HMO	1,274,708 (55.4%)	990,497 (81.7%)	0.59
PPO	1,024,910 (44.6%)	221,345 (18.3%)	0.59
**MA Plan Payer, N (%)**			
UnitedHealth	833,951 (36.3%)	66,902 (5.5%)	0.82
Humana	491,848 (21.4%)	52,488 (4.3%)	0.53
CVS	220,000 (9.6%)	89,987 (7.4%)	0.08
CIGNA	50,249 (2.2%)	1,115 (0.1%)	0.20
BCBS Association	150,140 (6.5%)	33,392 (2.8%)	0.18
Centene	36,327 (1.6%)	75,615 (6.2%)	0.24
Anthem	9,938 (0.4%)	128,095 (10.6%)	0.46
All Others	507,165 (22.1%)	764,248 (63.1%)	0.91

*Excluded MA beneficiaries include those enrolled in MA contracts deemed unreliable for research (1,095,894) and those who were not enrolled in MA for all months alive during the year (115,894).

eTable 2. Patient Characteristics of Medicare Advantage Beneficiaries, Overall and by Payer

	MA Overall	United Health	Humana	CVS	CIGNA	BCBS Association	Centene	Anthem	Other Payers
**Total Patients**	2,299,618	833,951	491,848	220,000	50,249	150,140	36,327	9,938	507,165
**Age, Mean (SD)**	75.3 (7.3)	75.5 (7.4)	75.1 (7.0)	75.6 (7.5)	76.1 (7.1)	75.2 (7.2)	74.3 (6.9)	74.4 (7.0)	75.2 (7.2)
**Age Group**									
65-74	53.1%	52.6%	53.3%	51.7%	48.1%	53.8%	58.7%	58.0%	54.0%
75-84	34.0%	33.5%	35.3%	34.0%	38.0%	33.9%	31.7%	31.9%	33.5%
85+	12.9%	13.8%	11.4%	14.3%	14.0%	12.3%	9.6%	10.1%	12.5%
**Sex, %**									
Female	42.8%	41.8%	42.7%	45.0%	42.4%	44.8%	42.0%	44.6%	42.9%
Male	57.2%	58.2%	57.3%	55.0%	57.6%	55.2%	58.0%	55.4%	57.1%
**Race/Ethnicity, %**									
Non-Hispanic Asian	3.3%	3.6%	2.0%	2.3%	2.7%	0.8%	9.0%	1.6%	4.9%
Non-Hispanic Black	12.1%	11.7%	14.2%	11.6%	19.6%	9.1%	38.3%	16.5%	9.4%
Hispanic	9.8%	10.0%	7.2%	4.7%	35.1%	1.8%	16.9%	59.7%	12.6%
Non-Hispanic White	72.6%	72.4%	74.8%	79.1%	41.5%	85.9%	34.3%	21.0%	70.6%
Other/Unknown	2.2%	2.3%	1.8%	2.4%	1.1%	2.4%	1.4%	1.1%	2.5%
**Dual Eligibility, %**									
Medicare & Medicaid Eligible	13.4%	14.1%	11.2%	4.3%	32.0%	2.9%	41.1%	53.3%	16.7%
Medicare Only	86.6%	85.9%	88.8%	95.8%	68.0%	97.1%	58.9%	46.7%	83.3%
**Low Income Subsidy, %**									
Non-LIS Recipient	83.0%	82.7%	84.6%	93.7%	61.4%	95.3%	51.8%	44.5%	78.9%
Partial LIS Recipient	16.0%	16.4%	14.1%	5.7%	36.8%	4.2%	46.4%	54.5%	20.1%
Full LIS Recipient	1.0%	0.9%	1.2%	0.5%	1.7%	0.5%	1.9%	1.0%	1.0%
**HCC Risk Score, Mean (SD)**	0.92 (1.0)	0.93 (1.0)	0.92 (1.0)	0.90 (1.0)	0.94 (1.0)	0.90 (1.0)	1.00 (1.1)	0.85 (0.9)	0.91 (1.0)
**Locality, %**									
Urban	96.8%	97.0%	94.9%	97.7%	99.4%	94.0%	97.0%	99.7%	98.3%
Rural	3.2%	3.0%	5.1%	2.3%	0.6%	6.0%	3.0%	0.3%	1.7%
**Census Region, %**									
Northeast	21.0%	17.7%	18.8%	26.3%	3.7%	58.9%	6.2%	0.5%	18.2%
Midwest	16.7%	14.1%	2.8%	29.3%	11.8%	11.4%	4.6%	1.8%	32.2%
South	42.8%	43.7%	69.8%	37.1%	68.6%	22.3%	74.3%	81.0%	18.1%
West	19.5%	24.5%	8.7%	7.3%	15.9%	7.4%	14.9%	16.8%	31.4%
**Area Deprivation Index, Mean (SD)**	65.2 (18.6)	61.8 (20.5)	64.4 (17.6)	62.8 (20.3)	65.8 (15.7)	70.7 (15.6)	64.7 (19.4)	40.0 (16.4)	66.0 (17.5)

© 2024 Duggan C et al. *JAMA Network Open.*

eTable 3. Adjusted Rates Across Specific Low-Value Services per 100 Beneficiary-Years in Traditional Medicare and Medicare Advantage

Low-Value Services	Medicare Advantage	Traditional Medicare	Absolute difference (95% CI)	*P* value
**Total Low-Value Services Among Eligible Sample**	50.02	52.48	-2.46 (-3.16, -1.75)	<0.001
**Common Treatments**	58.13	75.71	-17.58 (-19.31, -15.85)	<0.001
Antibiotics for Upper Respiratory/Ear Infections	59.72	60.53	-0.81 (-1.39, -0.22)	<0.01
Two or More Antipsychotic Medications	54.4	101.17	-46.77 (-51.36, -42.19)	<0.001
Antidepressants Monotherapy in Bipolar Disorder	9.8	9.02	0.78 (-0.12, 1.68)	0.08
**Imaging**	16.67	16.04	0.63 (0.14, 1.12)	0.01
Lower back Pain Image	0.89	0.87	0.01 (-0.03, 0.06)	0.50
Headache Image	5.44	5.4	0.05 (-0.24, 0.33)	0.75
Syncope Image	3.51	3.12	0.39 (0.14, 0.65)	<0.01
Electroencephalography (EEG) for Headaches	0.55	0.56	-0.01 (-0.07, 0.05)	0.76
Imaging of the Carotid Arteries	0.49	0.58	-0.09 (-0.13, -0.06)	<0.001
CT Head/Brain for Sudden Hearing Loss	1.91	1.86	0.06 (-0.06, 0.17)	0.34
Imaging for Uncomplicated Acute Rhinosinusitis	0.95	0.99	-0.04 (-0.11, 0.02)	0.21
ED CT Scans For Dizziness	11.98	11.87	0.11 (-0.39, 0.62)	0.66
Imaging Tests for Eye Disease	48.77	43.12	5.65 (4.53, 6.77)	<0.001
X-ray for Diagnosis of Plantar Fasciitis/Heel Pain	8.54	7.64	0.90 (0.31, 1.48)	<0.01
MRI for Rheumatoid Arthritis	1.92	2.94	-1.02 (-1.90, -0.15)	0.03
**Diagnostic and Preventative Testing**	9.03	6.47	2.56 (2.11, 3.01)	<0.001
Immunoglobulin G / immunoglobulin E testing	0.25	0.3	-0.05 (-0.07, -0.03)	<0.001
Diagnostics Chronic Urticaria	6.82	6.89	-0.07 (-2.40, 2.25)	0.95
Total or Free T3 Level	6.98	7.92	-0.94 (-1.33, -0.54)	<0.001
PTH for CKD	10.61	11.2	-0.59 (-1.00, -0.18)	<0.01
DEXA	0.11	0.09	0.02 (0.00, 0.04)	0.05
25-OH-Vitamin D Deficiency Screening	12.81	7.5	5.31 (4.26, 6.36)	<0.001
**Cardiovascular Testing**	8.49	9.35	-0.87 (-1.09, -0.64)	<0.001
Coronary Artery Calcium Scoring for Known CAD	0.06	0.07	-0.01 (-0.03, 0.01)	0.19
Cardiac Stress Testing	6.72	7.24	-0.52 (-0.80, -0.24)	<0.001
EKGs and Other Cardiac Screens	8.53	9.37	-0.84 (-1.08, -0.61)	<0.001
Coronary Angiography	1.69	1.95	-0.27 (-0.43, -0.10)	<0.01
**Procedures and Surgeries**	1.21	1.37	-0.16 (-0.24, -0.08)	<0.001
Arthroscopic Lavage and Debridement for Knee OA	0.12	0.1	0.02 (-0.01, 0.05)	0.13
Renal Artery Revascularization	7.78	11.42	-3.64 (-4.66, -2.62)	<0.001
Vertebroplasty	6.59	8.26	-1.67 (-2.21, -1.13)	<0.001
Proton Beam Therapy for Prostate Cancer	0.14	0.27	-0.13 (-0.21, -0.05)	<0.01
**Preoperative Evaluation**	8.86	9.62	-0.76 (-0.89, -0.64)	<0.001
Preoperative Baseline Laboratory Studies	11.81	12.46	-0.65 (-0.81, -0.49)	<0.001
Preop Cardiac Echocardiography or Stress Testing	0.03	0.04	-0.01 (-0.01, 0.00)	0.02
Preoperative EKG, Chest X-ray and PFT	1.2	1.26	-0.05 (-0.11, 0.00)	0.04
PFT Prior to Cardiac Surgery	2.21	2.12	0.09 (-0.33, 0.51)	0.67
**Cancer Screening**	13.38	13.88	-0.50 (-0.78, -0.21)	<0.001
PSA	30.12	30.71	-0.58 (-1.14, -0.02)	0.04
Colorectal Cancer Screening	9.68	8.52	1.16 (0.69, 1.63)	<0.001
Cervical Cancer Screening	3.06	3.75	-0.69 (-0.85, -0.53)	<0.001

Notes: Over-dispersed Poisson regression models were used and adjusted for age, sex, race/ethnicity, dual eligibility status, and CMS Hierarchical Condition Category (HCC) risk score (with an 11% deflation of HCC risk score among MA beneficiaries). Models also included HRR fixed effects and offsets for beneficiaries who died during the year.

© 2024 Duggan C et al. *JAMA Network Open.*

eTable 4. Adjusted Rates Across Specific Low-Value Services per 100 Beneficiary-Years Among MA Beneficiaries Enrolled in HMOs versus PPOs

Low-Value Services	Rate Per 100 Beneficiary-Years			
	MA HMO Plans	MA PPO Plans	Difference, HMO-PPO (95% CI)	*P* value
**Total Low-Value Services Among Eligible Population**	48.03	52.66	-4.63 (-5.53, -3.74)	<0.001
**Common Treatments**	53.82	58.74	-4.92 (-6.97, -2.87)	<0.001
Antibiotics for Acute Upper Respiratory and Ear Infections	59.24	62.33	-3.09 (-4.00, -2.18)	<0.001
Two or More Antipsychotic Medications	41.62	50.65	-9.03 (-15.53, -2.53)	<0.01
Antidepressants Monotherapy in Bipolar Disorder	10.36	9.94	0.42 (-0.91, 0.75)	0.53
**Imaging**	15.39	17.56	-2.17 (-2.70, -1.64)	<0.001
Lower back Pain Image	1.03	0.86	0.17 (0.09, 0.25)	<0.001
Headache Image	6.09	5.18	0.91 (0.51, 1.31)	<0.001
Syncope Image	3.77	3.37	0.40 (-0.08, 0.87)	0.10
Electroencephalography (EEG) for Headaches	0.62	0.49	0.13 (0.03, 0.24)	0.02
Imaging of the Carotid Arteries	0.47	0.54	-0.07 (-0.11, -0.04)	<0.001
CT Head/Brain for Sudden Hearing Loss	1.89	1.7	0.19 (0.00, 0AI.38)	0.05
Imaging for Uncomplicated Acute Rhinosinusitis	0.92	0.96	-0.04 (-0.15, 0.07)	0.47
ED CT Scans For Dizziness	12.47	12.75	-0.28 (-1.29, 0.72)	0.58
Imaging Tests for Eye Disease	46.87	48.63	-1.75 (-3.03, -0.48)	<0.01
X-ray for Diagnosis of Plantar Fasciitis/Heel Pain	9.39	8.09	1.30 (0.36, 2.24)	<0.01
MRI for Rheumatoid Arthritis	1.89	1.98	-0.08 (-1.77, 1.60)	0.92
**Diagnostic and Preventative Testing**	9.73	8.85	0.88 (0.46, 1.30)	<0.001
Immunoglobulin G / immunoglobulin E testing	0.25	0.26	-0.01 (-0.06, 0.04)	0.62
Diagnostics Chronic Urticaria	6.35	8.81	-2.46 (-6.65, 0.73)	0.23
Total or Free T3 Level	6.48	7.89	-1.41 (-1.91, -0.92)	<0.001
PTH for CKD	9.83	11.14	-1.31 (-1.91, -0.71)	<0.001
DEXA	0.12	0.11	0.01 (-0.02, 0.04)	0.59
25-OH-Vitamin D Deficiency Screening	14.85	11.5	3.35 (02.59, 4.12)	<0.001
**Cardiovascular Testing**	8.57	8.66	-0.08 (-0.33, 0.16)	0.49
Coronary Artery Calcium Scoring for Known CAD	0.05	0.06	-0.01 (-0.03, 0.01)	0.17
Cardiac Stress Testing	6.45	6.89	-0.44 (-0.87, -0.01)	0.04
EKGs and Other Cardiac Screens	8.61	8.61	0.00 (-0.24, 0.25)	0.99
Coronary Angiography	1.81	1.89	-0.08 (-0.39, 0.22)	0.60
**Procedures and Surgeries**	1.04	1.12	-0.08 (-0.24, 0.08)	0.34
Arthroscopic Lavage and Debridement for Knee OA	0.14	0.09	0.04 (0.00, 0.08)	0.06
Renal Artery Revascularization	7.53	7.18	0.35 (-1.42, 0.12)	0.70
Vertebroplasty	5.8	6.94	-1.15 (-2.29, -0.01)	0.05
Proton Beam Therapy for Prostate Cancer	0.11	0.15	-0.04 (-0.14, 0.06)	0.45
**Preoperative Evaluation**	8.57	9.1	-0.52 (-0.71, -0.34)	<0.001
Preoperative Baseline Laboratory Studies	11.5	11.98	-0.48 (-0.72, -0.24)	<0.001
Preop Cardiac Echocardiography or Stress Testing	0.03	0.03	0.00 (-0.01, 0.01)	0.63
Preoperative EKG, Chest X ray and PFT	1.21	1.16	0.05 (-0.02, 0.12)	0.16
PFT prior to Cardiac Surgery	2.68	1.86	0.82 (0.05, 0.59)	0.04
**Cancer Screening**	13.62	14.89	-1.28 (-1.65, -0.90)	<0.001
PSA	30.32	32.01	-1.69 (-2.42, -0.97)	<0.001
Colorectal Cancer Screening	9.22	8.55	0.67 (-0.01, 0.36)	0.06
Cervical Cancer Screening	2.73	3.88	-1.15 (-1.31, -0.99)	<0.001

© 2024 Duggan C et al. *JAMA Network Open.*

eFigure. Adjusted Rates of LVS Per 100 Beneficiary-Years in Traditional Medicare vs. Medicare Advantage by Major Payer

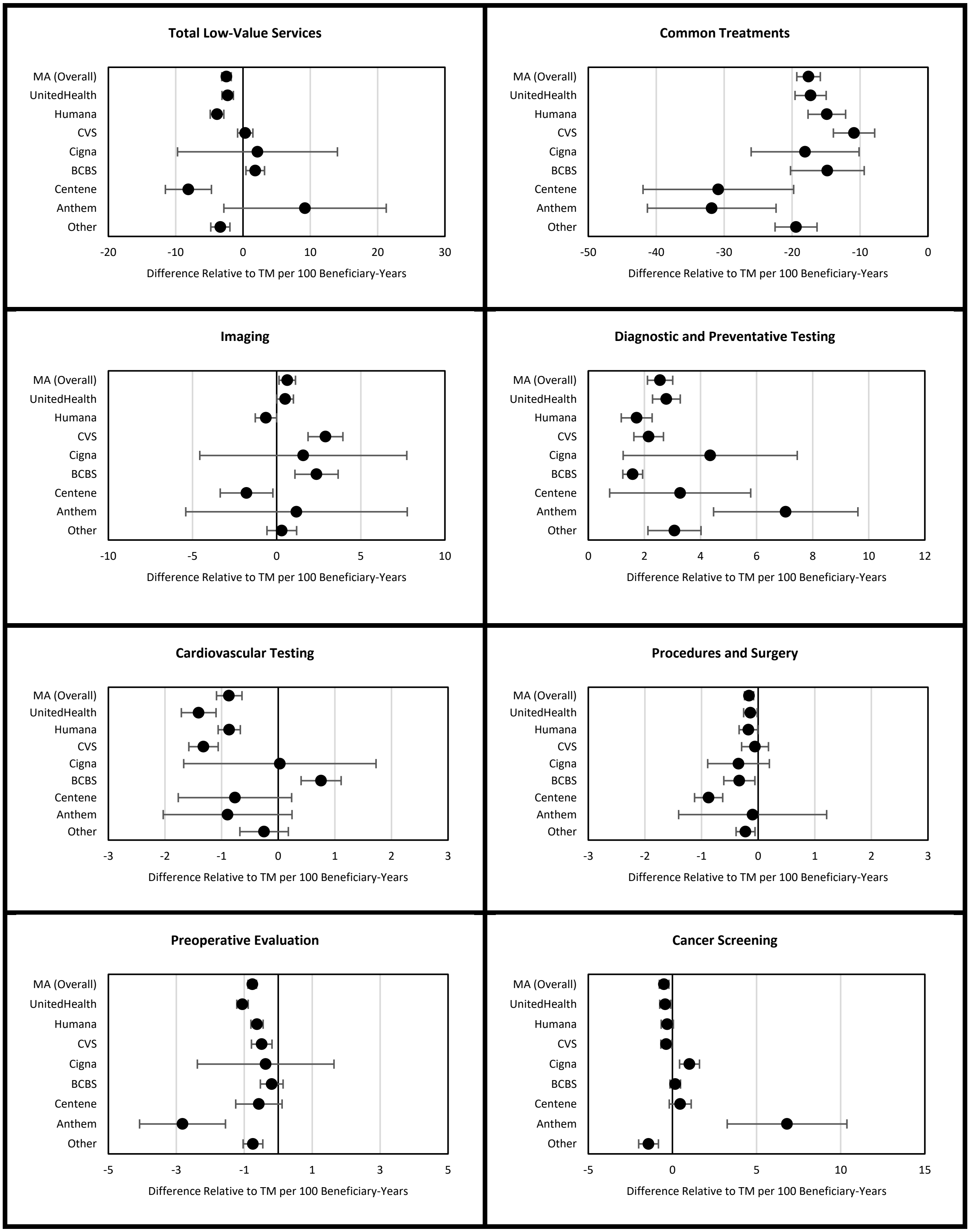

© 2024 Duggan C et al. *JAMA Network Open.*

eTable 5. Unadjusted and Adjusted Rates of LVS per 100 Beneficiary-Years Traditional Medicare and Medicare Advantage

	0% Reduced Risk Scores		6% Reduced Risk Scores		16% Reduced Risk Scores	
	Absolute Difference (95% CI), MA-TM	P value	Absolute Difference (95% CI), MA-TM	P value	Absolute Difference (95% CI), MA-TM	P value
Total LVS Among Eligible Population	-2.39 (-2.62, -2.17)	<0.001	-2.50 (-2.72, -2.27)	<0.001	-2.67 (-2.90, -2.45)	<0.001
Common Treatments	-17.12 (-18.41, -15.83)	<0.001	-17.61 (-18.90, -16.32)	<0.001	-18.19 (-19.48, -16.90)	<0.001
Imaging	0.43 (0.31, 0.54)	<0.001	0.61 (0.50, 0.72)	<0.001	0.78 (0.67, 0.89)	<0.001
Diagnostic and Preventative Testing	2.55 (2.47, 2.62)	<0.001	2.56 (2.48, 2.63)	<0.001	2.55 (2.47, 2.63)	<0.001
Cardiovascular Testing	-0.55 (-0.62, -0.48)	<0.001	-0.84 (-0.91, -0.77)	<0.001	-1.15 (-1.21, -1.08)	<0.001
Procedures and Surgeries	-0.26 (-0.34, -0.17)	<0.001	-0.17 (-0.26, -0.08)	<0.001	-0.11 (-0.20, -0.02)	0.016
Preoperative Evaluation	-0.60 (-0.68, -0.51)	<0.001	-0.74 (-0.83, -0.66)	<0.001	-0.90 (-0.99, -0.81)	<0.001
Cancer Screening	-0.40 (-0.47, -0.33)	<0.001	-0.50 (-0.57, -0.43)	<0.001	-0.72 (-0.79, -0.65)	<0.001

© 2024 Duggan C et al. *JAMA Network Open.*

eTable 6. Adjusted Rates of LVS per 100 Beneficiary-Years in Traditional Medicare vs. Medicare Advantage, Including Beneficiaries Who Switched During the Year

Low-Value Services (LVS)	Adjusted Rate Per 100 Beneficiary-Years			
	Started in MA	Started in TM	Adjusted Absolute Difference (95% CI)	*P* value
**Total LVS Among Eligible Sample**	51.21	52.85	-1.64 (-2.35, -0.93)	<0.001
**Common Treatments**	60.59	76.74	-16.15 (-17.89, -14.42)	<0.001
**Imaging**	16.77	16.09	0.68 (0.19, 1.17)	<0.01
**Diagnostic and Preventative Testing**	8.87	6.58	2.28 (1.80, 2.77)	<0.001
**Cardiovascular Testing**	9.48	9.4	0.09 (-0.10, 0.28)	0.37
**Procedures and Surgeries**	1.35	1.46	-0.11 (-0.20, -0.02)	0.02
**Preoperative Evaluation**	9.34	9.68	-0.34 (-0.46, -0.22)	<0.001
**Cancer Screening**	13.41	13.86	-0.44 (-0.71, -0.17)	<0.01

MA = Medicare Advantage; TM = Traditional Medicare. Sample includes beneficiaries who switched between MA and TM during the year. Beneficiaries were assigned to MA or TM based on their enrollment at the start of 2018. The final sample includes 121,812 switchers; 28,227 started in MA and 93,585 started in TM. Over-dispersed Poisson regression models were used and adjusted for age, sex, race/ethnicity, dual eligibility status, and CMS Hierarchical Condition Category (HCC) risk score (with an 11% deflation of HCC risk score among MA beneficiaries). Models also included HRR fixed effects and offsets for beneficiaries who died during the year.

© 2024 Duggan C et al. *JAMA Network Open.*

eTable 7. Adjusted Rates of LVS per 100 Beneficiary-Years in Medicare Advantage, Comparison of Included vs Excluded MA Plans

**Low-Value Services (LVS)**	**Adjusted Rate Per 100 Beneficiary-Years**			
	**Included MA Plans**	**Excluded MA Plans**	**Adjusted Absolute Difference (95% CI)**	***P* value**
**Total LVS Among Eligible Sample**	52.27	47.24	-5.03 (-7.04, -3.02)	<0.001
**Common Treatments**	71.18	55.48	-15.70 (-18.36, -13.03)	<0.001
**Imaging**	16.41	15.61	-0.80 (-1.74, 0.14)	<0.001
**Diagnostic and Preventative Testing**	7.61	9.31	1.70 (1.05, 2.36)	<0.001
**Cardiovascular Testing**	9.58	10.18	0.61 (0.26, 0.95)	0.21
**Procedures and Surgeries**	1.37	1.37	0.00 (-0.21, 0.21)	0.70
**Preoperative Evaluation**	9.63	9.71	0.08 (-0.17, 0.32)	<0.001
**Cancer Screening**	13.77	11.08	-2.69 (-3.81, -1.56)	<0.001

Notes: Over-dispersed Poisson regression models were used and adjusted for age, sex, race/ethnicity, dual eligibility status, and CMS Hierarchical Condition Category (HCC) risk score (with an 11% deflation of HCC risk score among MA beneficiaries). Models also included HRR fixed effects and offsets for beneficiaries who died during the year. Of note, adjusted rates for the MA beneficiaries enrolled in included plans should not necessarily match the estimates reported for MA beneficiaries in the primary analysis.

© 2024 Duggan C et al. *JAMA Network Open.*

eTable 8. Adjusted Rates of LVS per 100 Beneficiary-Years in Traditional Medicare vs. Medicare Advantage, Excluding Beneficiaries Who Died During the Year

Low-Value Services (LVS)	Adjusted Rate Per 100 Beneficiary-Years			
	Medicare Advantage	Traditional Medicare	Adjusted Absolute difference (95% CI)	*P* value
**Total LVS Among Eligible Sample**	51.73	53.53	-1.80 (-2.52, -1.09)	<0.001
**Common Treatments**	60.47	77.21	-16.74 (-18.52, -14.95)	<0.001
**Imaging**	17.06	16.31	0.75 (0.25, 1.25)	<0.01
**Diagnostic and Preventative Testing**	8.96	6.56	2.40 (1.89, 2.91)	<0.001
**Cardiovascular Testing**	9.58	9.56	0.01 (-0.19, 0.21)	0.90
**Procedures and Surgeries**	1.19	1.26	-0.07 (-0.15, 0.01)	0.10
**Preoperative Evaluation**	9.54	9.89	-0.34 (-0.47, -0.22)	<0.001
**Cancer Screening**	13.65	14.23	-0.58 (-0.88, -0.29)	<0.001

Notes: Over-dispersed Poisson regression models were used and adjusted for age, sex, race/ethnicity, dual eligibility status, and CMS Hierarchical Condition Category (HCC) risk score (with an 11% deflation of HCC risk score among MA beneficiaries). Models also included HRR fixed effects. Decedents were excluded from the sample. The sample of beneficiaries that survived all 12 months included 2,210,909 MA beneficiaries and 3,492,894 beneficiaries.

© 2024 Duggan C et al. *JAMA Network Open.*

eTable 9. Adjusted Rates of LVS per 100 Beneficiary-Years in BCBS Plans vs. TM

State	Total LVS Among Eligible Sample, Adjusted Rate Per 100 Beneficiary-Years			
	BCBS	TM	Adjusted Absolute Difference Relative to TM Beneficiaries in State (95% CI)	*P* value
**Michigan**	53.68	52.89	0.79 (-0.47, 2.05)	0.22
**Alabama**	57.57	53.26	4.31 (2.78, 5.85)	<0.001
**South Carolina**	61.81	53.19	8.62 (3.04, 14.20)	<0.01
**Arizona**	49.76	53.11	-3.34 (-6.65, -0.03)	0.05
**Rhode Island**	54.77	53.17	1.60 (1.31, 1.89)	<0.001

Notes: Over-dispersed Poisson regression models were used and adjusted for age, sex, race/ethnicity, dual eligibility status, and CMS Hierarchical Condition Category (HCC) risk score (with an 11% deflation of HCC risk score among MA beneficiaries). Models also included HRR fixed effects and offsets for beneficiaries who died during the year.

© 2024 Duggan C et al. *JAMA Network Open.*

eTable 10. Adjusted Rates of LVS per 100 Beneficiary-Years in Traditional Medicare vs. Medicare Advantage with County Fixed Effects

Low-Value Services (LVS)	Adjusted Rate Per 100 Beneficiary-Years			
	Medicare Advantage	Traditional Medicare	Adjusted Absolute Difference (95% CI)	*P* value
**Total LVS Among Eligible Sample**	52.76	51.11	-1.65 (-2.21, -1.09)	<0.001
**Common Treatments**	76.42	59.72	-16.70 (-18.36, -15.04)	<0.001
**Imaging**	16.12	16.81	0.69 (0.31, 1.07)	<0.001
**Diagnostic and Preventative Testing**	6.53	8.86	2.33 (1.90, 2.77)	<0.001
**Cardiovascular Testing**	9.37	9.52	0.14 (-0.04, 0.33)	<0.001
**Procedures and Surgeries**	1.47	1.32	-0.15 (-0.25, -0.05)	0.39
**Preoperative Evaluation**	9.65	9.4	-0.25 (-0.36, -0.14)	<0.001
**Cancer Screening**	13.92	13.48	-0.44 (-0.70, -0.19)	<0.001

Notes: Over-dispersed Poisson regression models were used and adjusted for age, sex, race/ethnicity, dual eligibility status, and CMS Hierarchical Condition Category (HCC) risk score (with an 11% deflation of HCC risk score among MA beneficiaries). Models also included county fixed effects and offsets for beneficiaries who died during the year.

© 2024 Duggan C et al. *JAMA Network Open.*
