# Supplementary material for: Evaluation of Low-Value Services Across Major Medicare Advantage Insurers and Traditional Medicare
Source: JAMA Netw Open. 2024 Nov 1;7(11):e2442633. doi: 10.1001/jamanetworkopen.2024.42633 (PMC11530944; doi:10.1001/jamanetworkopen.2024.42633)
Supplement: Supplement 2. — Data Sharing Statement [file jamanetwopen-e2442633-s002.pdf]

Data Sharing Statement

Duggan. Evaluation of Low-Value Services Across Major Medicare Advantage Insurers and Traditional Medicare. *JAMA Netw Open*. Published November 01, 2024. doi:10.1001/jamanetworkopen.2024.42633

Data
Data available: No

Additional Information
Explanation for why data not available: The Medicare data files used in this study may not be shared due to restrictions in our data use agreement. However, any researcher interested in Medicare data can obtain these data directly through Medicare and ResDAC.
